# Abl Family Kinases Regulate Endothelial Barrier Function *In Vitro* and in Mice

**DOI:** 10.1371/journal.pone.0085231

**Published:** 2013-12-19

**Authors:** Elizabeth M. Chislock, Ann Marie Pendergast

**Affiliations:** Department of Pharmacology and Cancer Biology, Duke University School of Medicine, Durham, North Carolina, United States of America; Cardiological Center Monzino, Italy

## Abstract

The maintenance of endothelial barrier function is essential for normal physiology, and increased vascular permeability is a feature of a wide variety of pathological conditions, leading to complications including edema and tissue damage. Use of the pharmacological inhibitor imatinib, which targets the Abl family of non-receptor tyrosine kinases (Abl and Arg), as well as other tyrosine kinases including the platelet-derived growth factor receptor (PDGFR), Kit, colony stimulating factor 1 receptor (CSF1R), and discoidin domain receptors, has shown protective effects in animal models of inflammation, sepsis, and other pathologies characterized by enhanced vascular permeability. However, the imatinib targets involved in modulation of vascular permeability have not been well-characterized, as imatinib inhibits multiple tyrosine kinases not only in endothelial cells and pericytes but also immune cells important for disorders associated with pathological inflammation and abnormal vascular permeability. In this work we employ endothelial *Abl* knockout mice to show for the first time a direct role for Abl in the regulation of vascular permeability *in vivo*. Using both Abl/Arg-specific pharmacological inhibition and endothelial *Abl* knockout mice, we demonstrate a requirement for Abl kinase activity in the induction of endothelial permeability by vascular endothelial growth factor both *in vitro* and *in vivo*. Notably, Abl kinase inhibition also impaired endothelial permeability in response to the inflammatory mediators thrombin and histamine. Mechanistically, we show that loss of Abl kinase activity was accompanied by activation of the barrier-stabilizing GTPases Rac1 and Rap1, as well as inhibition of agonist-induced Ca^2+^ mobilization and generation of acto-myosin contractility. In all, these findings suggest that pharmacological targeting of the Abl kinases may be capable of inhibiting endothelial permeability induced by a broad range of agonists and that use of Abl kinase inhibitors may have potential for the treatment of disorders involving pathological vascular leakage.

## Introduction

The endothelium forms a critical semi-permeable barrier between tissues and the bloodstream, regulating the transport of solutes and immune cells into and out of the circulation. The maintenance of this barrier is a dynamic and tightly-controlled process. Loosening of the endothelial barrier is induced by a variety of soluble factors, including cytokines and other inflammatory mediators, as well as vascular endothelial growth factor (VEGF), and is an important aspect of both normal angiogenic remodeling and inflammatory responses [1,2]. However, abnormally elevated vascular permeability is a key feature of a variety of pathological conditions, including cancer, sepsis, and ischemia-reperfusion injury [3,4]. This uncontrolled vascular leakage can lead to edema, increased interstitial fluid pressure, and tissue damage [4]. 

Vascular permeability can occur through both transcellular and paracellular mechanisms. In the transcellular pathway, solutes or cells pass through individual endothelial cells via vesicular transport mechanisms [5]. In contrast, paracellular permeability requires the dynamic opening and closure of inter-endothelial cell-cell adherens and tight junctions, enabling the passage of plasma molecules or cells between neighboring endothelial cells [6]. The transmembrane protein vascular endothelial cadherin (VE-cadherin) is the major structural component of endothelial adherens junctions and is a critical regulator of vascular integrity and endothelial barrier function [7]. Dimerization and clustering of VE-cadherin at sites of endothelial cell-cell contact leads to homotypic, Ca^2+^-dependent interaction of the extracellular domains of VE-cadherin proteins on neighboring cells, which are then linked indirectly to the actin cytoskeleton through the binding of the VE-cadherin intracellular domain to β-catenin and α-catenin proteins [8]. 

The endothelial adherens junction complex is targeted by a variety of vascular permeability-inducing factors, including VEGF, thrombin, and histamine. Stimulation of endothelial cells with these barrier-disruptive factors leads to dissolution of cell-cell junctions through mechanisms including VE-cadherin internalization, destabilization of adherens junction protein complexes, or reduced association of VE-cadherin complexes with the actin cytoskeleton [9–13]. In addition to direct effects on cell-cell junctions, endothelial barrier-disrupting factors increase acto-myosin contractility and centripetal tension, which weakens intercellular adhesion and can lead to cell retraction and formation of intercellular gaps [14,15]. Tyrosine phosphorylation has been implicated in the destabilization of the endothelial barrier by a variety of permeability-inducing factors. Increased tyrosine phosphorylation of adherens junction proteins including VE-cadherin and β-catenin has been observed following VEGF, histamine, and thrombin stimulation [9,16,17]; this phosphorylation has been linked to destabilization of cell-cell adhesion. Additionally, treatment with tyrosine kinase inhibitors decreased endothelial permeability induced by each of these agonists [9,14,18,19], demonstrating an important role for tyrosine kinases in the induction of endothelial barrier dysfunction.

The Abelson (Abl) family of non-receptor tyrosine kinases includes two members, Abl (Abl1) and Arg (Abl2), characterized by the presence of unique C-terminal actin-binding domains [20]. These kinases are activated downstream of a variety of growth factor and chemokine receptors, as well as following cadherin and integrin engagement, to regulate cellular responses including cytoskeletal remodeling, adhesion, and migration [20]. Our previous work has demonstrated a role for the Abl kinases in both formation and maintenance of epithelial adherens junctions [21], suggesting a potential role for these kinases in the regulation of barrier function. Interestingly, treatment with the Abl kinase pharmacological inhibitor imatinib (Gleevec) decreased interstitial fluid pressure in lung and colon cancer models, resulting in improved tumor oxygenation and drug delivery [22–24]. Imatinib treatment also reduced permeability following administration of thrombolytic tissue plasminogen activator in a murine model of ischemic stroke [25], suggesting a beneficial effect of imatinib on vascular barrier function. Pre-treatment with imatinib (or the more potent Abl kinase inhibitor nilotinib) similarly protected against pulmonary edema following lipopolysaccharide-induced acute lung injury in mice [26]. These protective effects have been attributed to the inhibition of the platelet-derived growth factor receptor (PDGFR), which is also targeted by imatinib [27]. However, recent studies have implicated the Abl kinases in the regulation of endothelial barrier function [28,29]. Expression of the Abl kinase is required for the endothelial barrier-promoting effects of sphingosine-1-phosphate *in vitro* [29]. Imatinib treatment protected against vascular leakage and edema in a murine sepsis model, which was attributed to the inhibition of the endothelial Arg kinase [28]. However, the *in vivo* protective effects of imatinib may result from inhibition of multiple tyrosine kinases and targeting of cell types other than endothelial cells, including immune cells.

In the current study, we demonstrate a requirement for activation of the Abl kinases in endothelial permeability induced by VEGF and the inflammatory mediators thrombin and histamine. Use of Abl/Arg-specific pharmacological inhibitors or *Abl* knockdown impaired induction of endothelial permeability in response to these agonists *in vitro*. VEGF-induced permeability similarly was decreased following Abl kinase inhibition *in vivo*. Importantly, impaired VEGF-induced permeability was also observed in conditional knockout mice lacking endothelial *Abl* expression. Mechanistically, we demonstrate that Abl kinase inhibition both increased activation of the endothelial barrier-supporting GTPases Rac1 and Rap1 and decreased the activation of pathways regulating induction of acto-myosin contractility in response to permeability-inducing factors. Taken together, these findings demonstrate an important role for the Abl kinases in mediating endothelial barrier dysfunction induced by a variety of agonists and support the potential use of Abl kinase inhibitors in the treatment of disorders characterized by pathological vascular permeability.

## Results

### Abl Kinases Are Activated Following Treatment with Endothelial Permeability-Inducing Factors

**Figure 1:**
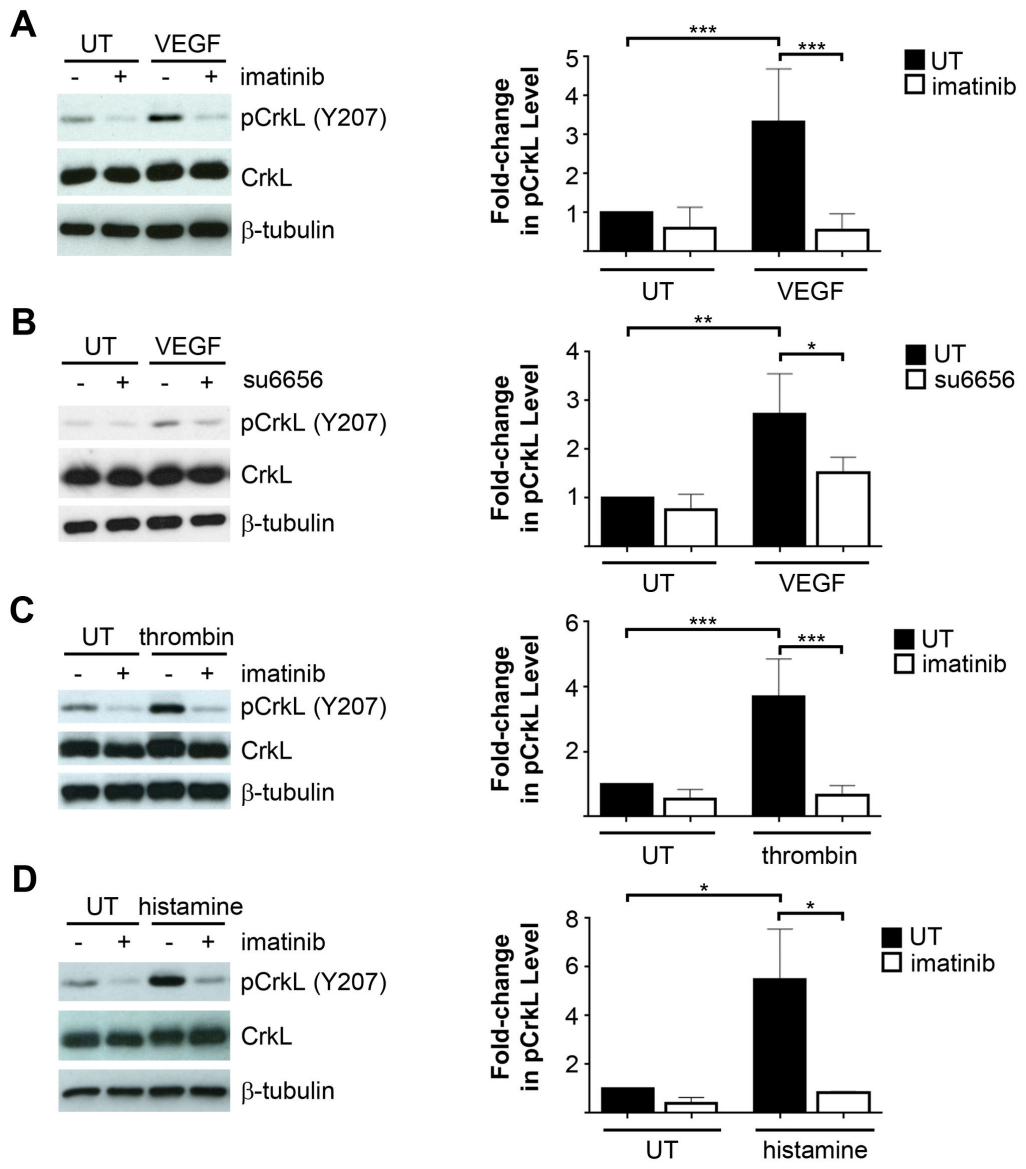
Abl kinases are activated following treatment with endothelial permeability-inducing factors. (**A**) Assessment of Abl kinase activation, as determined by phospho-CrkL tyrosine (Y) 207 levels, following stimulation of serum-starved HMVECs with 100ng/mL VEGF for 5 minutes, with or without imatinib pre-treatment (10μM). pCrkL (Y207) levels (normalized to total CrkL) are quantified in the right panel, relative to levels in untreated (UT) cells. Data are presented as means +/- SD (n=7). (**B**) Evaluation of pCrkL (Y207) levels in HMVECs treated with VEGF, with or without su6656 pre-treatment (1μM). pCrkL levels (normalized to total CrkL) are quantified in the right panel, relative to levels in untreated (UT) cells. Data are presented as means +/- SD (n=2). (**C**) Evaluation of Abl kinase activation (pCrkL Y207) following treatment of HMVECs with thrombin (1U/mL, 5 minutes), with or without imatinib pre-treatment. pCrkL levels (normalized to total CrkL) are quantified in the right panel, relative to levels in untreated (UT) cells. Data are presented as means +/- SD (n=5). (**D**) Assessment of Abl kinase activation (pCrkL Y207) following stimulation of HMVECs with histamine (100μM, 5 minutes), with or without imatinib pre-treatment. pCrkL levels (normalized to total CrkL) are quantified in the right panel, relative to levels in untreated (UT) cells. Data are presented as means +/- SD (n=3). *P<0.05; **P<0.01; ***P<0.001.

Endothelial barrier dysfunction can be induced in response to a variety of soluble mediators [2]. To assess a potential role for the Abl kinases in the regulation of endothelial barrier function, we initially evaluated Abl kinase activity following treatment of human microvascular endothelial cells (HMVECs) with the permeability-inducing factors VEGF, thrombin, and histamine. In agreement with previous findings in human umbilical vein endothelial cells (HUVECs) [28,30,31], stimulation of HMVECs with VEGF resulted in Abl kinase activation, as assessed by the phosphorylation of CrkL at tyrosine (Y) 207, an Abl-specific phosphorylation site [32] (Figure **1A**), which was prevented by pre-treatment with the ATP-competitive Abl kinase inhibitor imatinib. Interestingly, pre-treatment with the Src kinase inhibitor su6656 partially blocked Abl kinase activation in response to VEGF stimulation (Figure **1B**), suggesting that the Abl kinases may act downstream of Src family kinases in VEGF-mediated signaling. Imatinib treatment did not inhibit VEGF-induced tyrosine phosphorylation of Src family kinases (Figure **S1A**) or downstream phosphorylation of the Src targets FAK and paxillin [33,34] (Figure **S1B**), demonstrating that Abl kinase activity is not required for Src activation. Notably, Abl kinases were markedly activated by treatment of HMVECs with thrombin (Figure **1C**) or histamine (Figure **1D**). Thus, these findings demonstrate that the Abl kinases are activated in response to several distinct endothelial permeability-inducing mediators, suggesting a potential function for these kinases in mediating downstream permeability responses.

### Loss of Abl Kinase Function Decreased Endothelial Barrier Dysfunction In Vitro

**Figure 2:**
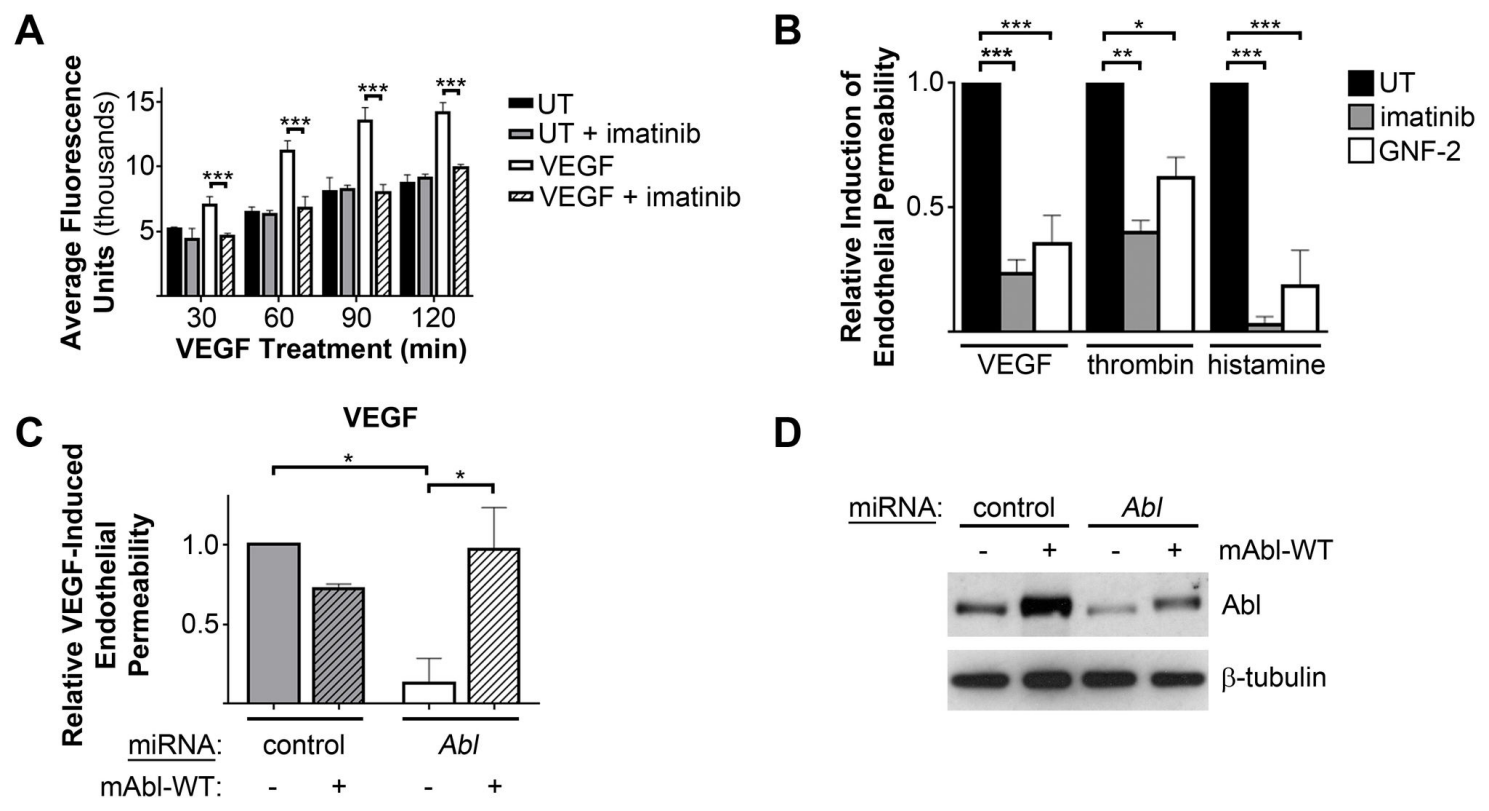
Loss of Abl kinase function decreased endothelial barrier dysfunction *in* *vitro*. (**A**) Evaluation of endothelial monolayer permeability, as assessed by passage of fluorescein-labeled dextran (molecular weight 40kDa) through HMVEC monolayers grown on Transwells, following treatment with VEGF (100ng/mL) with or without imatinib pre-treatment (10μM). Data shown are mean fluorescence of samples collected from bottom Transwell chambers at the indicated times post-VEGF treatment, +/- SD of three replicates per treatment. Data are representative of 3 independent experiments. (**B**) Quantification of inhibition of endothelial monolayer permeability to fluorescein-labeled dextran by imatinib (10μM) or GNF-2 (15μM). Endothelial barrier dysfunction was induced by treatment of HMVECs with VEGF (100ng/mL, 120 minutes), thrombin (1U/mL, 30 minutes) or histamine (100μM, 60 minutes). Values are expressed relative to permeability induction in vehicle-treated cells (UT). Data are presented as means +/- SEM (n=3). (**C**) Quantification of VEGF-induced endothelial permeability of HMVECs expressing either control or Abl miRNAs, with or without re-expression of miRNA-resistant, wild-type murine Abl (mAbl-WT). Values are expressed relative to VEGF-induced permeability in control miRNA-expressing cells. Data are presented as means +/- SEM (n=3). (**D**) Assessment of Abl protein levels following miRNA expression in HMVECs, with or without re-expression of miRNA-resistant Abl. *P<0.05; **P<0.01; ***P<0.001.

We examined whether the Abl kinases may play a role in the induction of endothelial permeability *in vitro*, by assessing the passage of fluorescein-labeled dextran through HMVEC monolayers following pharmacological inhibition of the Abl kinases. Consistent with previous reports [28,35], Abl kinase inhibition with imatinib greatly decreased endothelial barrier dysfunction induced by VEGF (Figures 2A-B). Imatinib also inhibited permeability induced by thrombin and histamine (Figure **2B**). As imatinib also inhibits kinases other than Abl and Arg, including the receptors CSF1R (also known as c-Fms), PDGFR, Kit, and the discoidin domain receptors [27,36,37], we examined the effects of the allosteric Abl kinase inhibitor GNF-2 on endothelial permeability. GNF-2, which binds to the myristate-binding pocket in the kinase domain of Abl and Arg, displays greater target specificity than imatinib and is not known to inhibit any additional kinases [38,39]. Importantly, the Abl/Arg-specific inhibitor GNF-2 also decreased endothelial permeability induced by VEGF, thrombin, or histamine (Figure **2B**), suggesting that the preservation of endothelial barrier function upon imatinib treatment likely results from inhibition of the Abl family kinases. 

To directly evaluate whether Abl is implicated in the regulation of endothelial permeability, we depleted Abl expression in HMVECs. VEGF-induced endothelial permeability was inhibited by micro-RNA (miRNA)-mediated *Abl* knockdown (Figures **2C** and S2A,C), and permeability induction was restored by re-expression of miRNA-resistant Abl (Figures 2C-D). A similar reduction of thrombin-induced endothelial barrier dysfunction was observed upon *Abl* knockdown (Figures S2B-C). Interestingly, Abl kinase inhibition using either imatinib or GNF-2 more potently inhibited endothelial permeability responses than did *Abl* knockdown alone (Figure **2B** vs Figure **S2C**), suggesting that Arg may also contribute to VEGF- and thrombin-induced endothelial permeability. However, dual knockdown of both Abl and Arg kinases led to a nearly twofold increase in baseline permeability in unstimulated HMVEC monolayers (Figures S2D-E), which precluded the analysis of VEGF- and thrombin-induced permeability. These data are consistent with our finding that depletion of both Abl and Arg proteins induces dissolution of adherens junctions in epithelial cells [21]. In all, these results demonstrate a requirement for Abl kinase activity, as well as Abl expression, in endothelial barrier dysfunction induced by several permeability-inducing factors. 

### Abl Kinase Activity Is Required for VEGF-Induced Permeability In Vivo

**Figure 3:**
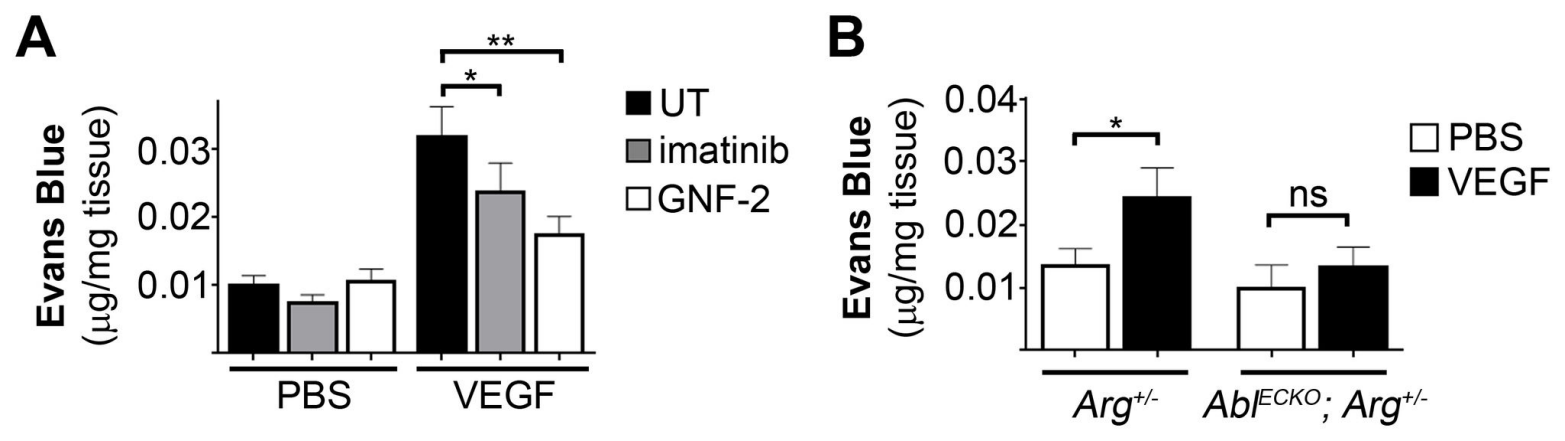
Abl kinases are required for VEGF-induced vascular permeability *in* *vivo*. (**A**) Evaluation of vascular leakage of Evans blue dye in mice following intradermal injection of VEGF (100ng, 15 minutes) with or without concomitant treatment with imatinib or GNF-2 (15μM). Dye extravasation was normalized to tissue weight. Values are presented as means +/- SD (n=12). (**B**) Quantification of VEGF-induced dermal vascular leakage of Evans blue dye in *Abl* ^*ECKO*^ *; Arg* ^*+/-*^ (Abl^flox/flox^; Arg^+/-^; Tie2-Cre^+/-^) and age/sex-matched Arg^+/-^ control mice (Abl^flox/flox^; Arg^+/-^; Tie2-Cre^-/-^). Dye extravasation was normalized to tissue weight. Values are presented as means +/- SD (Arg^+/-^ controls, n=8; *Abl* ^*ECKO*^ *; Arg* ^*+/-*^, n=6). *P<0.05; **P<0.01.

 To evaluate whether Abl kinases are involved in VEGF-induced vascular permeability *in vivo*, we employed both pharmacological inhibition and genetic inactivation of endothelial *Abl* kinase in mice. Consistent with our *in vitro* findings, inhibition of the Abl kinases with either imatinib or GNF-2 decreased VEGF-induced vascular leakage of albumin (by approximately 30% and 50%, respectively), as assessed by extravasation of intravenously-administered Evans blue dye following intradermal administration of VEGF (Figure **3A**). 

To directly assess the role of the Abl kinases in VEGF-induced permeability *in vivo*, we generated conditional knockout mice lacking Abl kinase expression in the endothelium, by crossing mice carrying a floxed *Abl* allele (*Abl* ^*flox/flox*^) on an *Arg* ^*-/-*^ background to *Tie2-Cre* mice [31]. As loss of both endothelial *Abl* and *Arg* expression (*Abl* ^*flox/flox*^ *; Arg* ^*-/-*^ *; Tie2-Cre* ^*+/-*^) resulted in late-stage embryonic and perinatal lethality [31], we instead examined permeability responses using endothelial *Abl* knockout mice on an *Arg* ^*+/-*^ background (*Abl* ^*flox/flox*^ *; Arg* ^*+/-*^ *; Tie2-Cre* ^*+/-*^, referred to as *Abl* ^*ECKO*^ *; Arg* ^*+/-*^), which survive to adulthood. Notably, VEGF-induced vascular permeability was reduced in *Abl* ^*ECKO*^ *; Arg* ^*+/-*^ mice (Figure **3B**). While VEGF induced a two-fold increase in Evans blue dye extravasation in *Arg* ^*+/-*^ control mice (*Abl* ^*flox/flox*^ *; Arg* ^*+/-*^ *; Tie2-Cre* ^*-/-*^), no significant increase in vascular leakage was observed following VEGF treatment in *Abl* ^*ECKO*^ *; Arg* ^*+/-*^ mice. A previous report suggested that Arg, rather than Abl, mediates the *in vitro* endothelial barrier-enhancing effects of imatinib [28]. However, our genetic results show that Abl is a critical player in the regulation of endothelial barrier function *in vivo*. Taken together, these findings demonstrate a requirement for the Abl family kinases in VEGF-induced vascular permeability *in vivo*.

### Abl Kinase Activity Is Required for VEGF- and Thrombin-Induced Disruption of Endothelial Adherens Junctions

**Figure 4:**
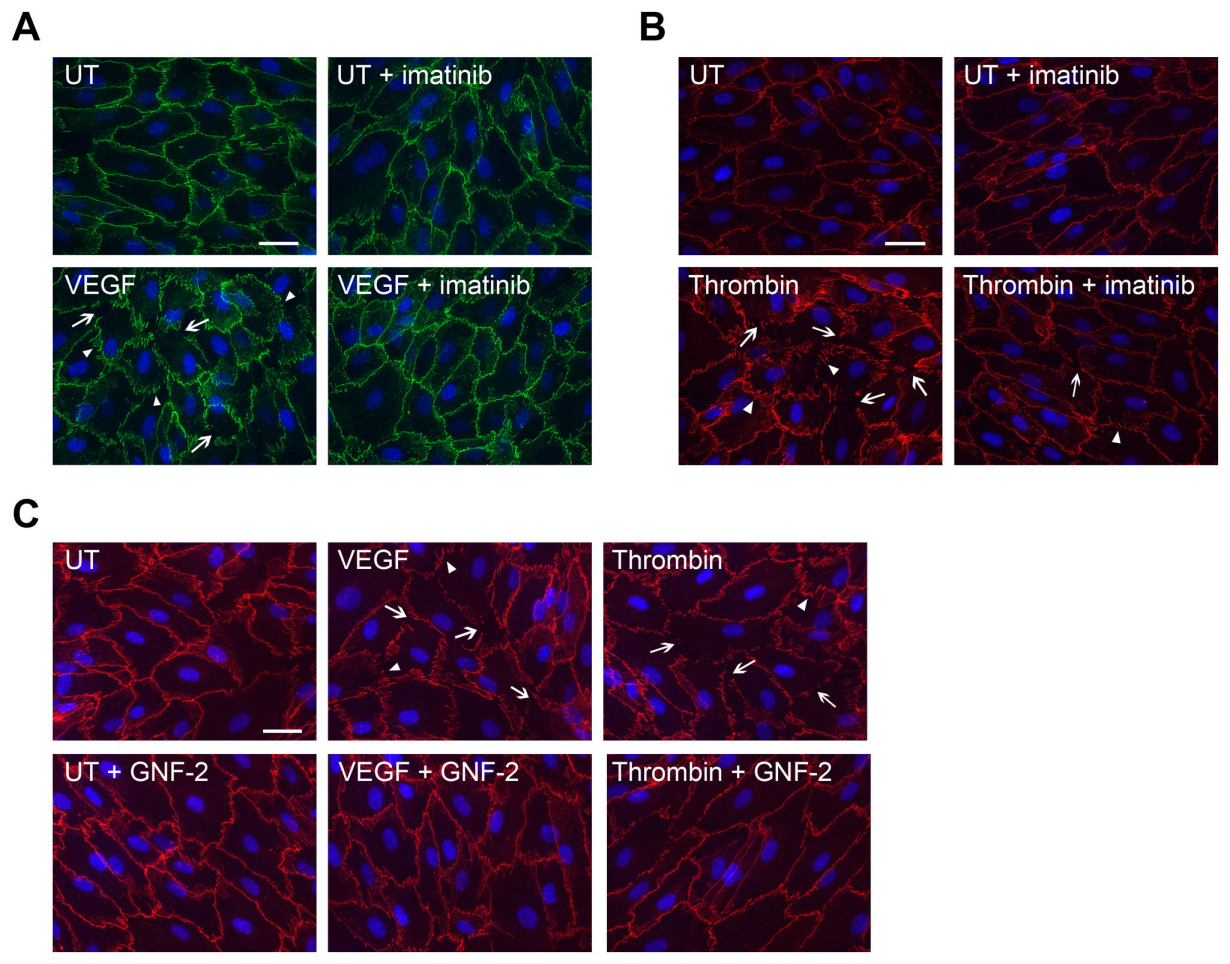
Abl kinase activity is required for VEGF- and thrombin-induced disruption of endothelial adherens junctions. (**A**) Staining of HMVEC monolayers for the adherens junction marker VE-cadherin (green) following treatment with VEGF (100ng/mL, 30 minutes), with or without imatinib pre-treatment (10μM). (**B**) VE-cadherin staining (red) of HMVEC monolayers treated with thrombin (1U/mL, 5 minutes), +/- imatinib. (**C**) VE-cadherin staining (red) of VEGF or thrombin-treated HMVECs, with or without GNF-2 pre-treatment (15μM). VEGF and thrombin treatment induced formation of inter-endothelial cell gaps (arrows) and destabilization of endothelial cell-cell junctions (“zig-zag” VE-cadherin staining pattern, arrowheads), which were reduced by pre-treatment with Abl kinase inhibitors.

Induction of endothelial barrier dysfunction has previously been linked to disruption of endothelial cell-cell adhesion, through the phosphorylation and disruption of endothelial adherens junction complexes, as well as VE-cadherin mislocalization and internalization [9–13]. As the Abl kinases are required for adherens junction formation and mediate signaling downstream of cadherin engagement in epithelial cells [21], we examined whether the Abl kinases might regulate VE-cadherin dynamics in endothelial cells following stimulation with permeability-inducing factors. While a continuous pattern of VE-cadherin staining was observed at endothelial cell-cell junctions in unstimulated cells, both VEGF and thrombin treatment disrupted VE-cadherin localization, leading to destabilization of endothelial adherens junctions (“zig-zag” VE-cadherin staining pattern, arrowheads) and formation of inter-endothelial cell gaps (arrows) (Figures 4A-C). Consistent with their anti-permeability effects, pre-treatment with the Abl kinase inhibitors imatinib (Figures 4A-B) or GNF-2 (Figure **4C**) reduced the VEGF- and thrombin-induced disruption of VE-cadherin localization. However, imatinib treatment did not alter VE-cadherin cell-surface levels (Figure **S3A**) or association with catenin proteins (Figures S3B-E). VEGF-induced endothelial permeability previously has been linked to tyrosine phosphorylation of adherens junction proteins including VE-cadherin and β-catenin, which is thought to destabilize cellular adherens junctions [6,10,16]. However, we did not observe changes in VE-cadherin or β-catenin tyrosine phosphorylation in response to VEGF stimulation, either in the presence or absence of imatinib treatment (data not shown). Taken together, these findings suggest that Abl kinase inhibition prevents VEGF- and thrombin-induced disruption of endothelial adherens junctions independently of direct effects on VE-cadherin and other adherens junction components.

Activation of endothelial nitric oxide synthase (eNOS) and resulting generation of nitric oxide (NO) following VEGF treatment has been shown to contribute to VEGF-induced endothelial permeability [19,40] through mechanisms including β-catenin S-nitrosylation [41] and regulation of actin cytoskeletal architecture [41,42]. While Abl kinase expression is required for lipopolysaccharide-induced NO production in macrophages [43], we did not observe any effect of Abl kinase inhibition on VEGF-induced eNOS (serine 1177) phosphorylation or NO production in endothelial cells (Figures S4A-B). Further, imatinib treatment protected against VEGF-induced endothelial barrier dysfunction even in the presence of the NO donor S-Nitroso-*N*-Acetylpenicillamine (SNAP) (Figure **S4C**), and VEGF-induced Abl kinase activation was not prevented by pre-treatment with the eNOS inhibitor L-NAME (Figure **S4D**). Taken together, these findings suggest that the activation of the Abl kinases and the anti-permeability effects of Abl kinase inhibition each are independent of VEGF-induced eNOS activation.

### Activation of Rac1 and Rap1 GTPases Following Abl Kinase Inhibition

Endothelial permeability and adherens junction stability are modulated by the activity of a variety of small guanosine triphosphatase (GTPase) proteins, which regulate cytoskeletal remodeling and act either to stabilize or disrupt barrier function [44]. Of these, the Rho family GTPase Rac1 and Ras family GTPase Rap1 have been identified as important mediators in the maintenance of endothelial barrier function. Rac1 activation opposes the induction of endothelial permeability, in part by remodeling of cortical actin and stabilizing adherens junctions [45–47]. Consistent with previously-reported findings [28], Abl kinase inhibition with imatinib increased the levels of active, GTP-bound Rac1 both in unstimulated and VEGF-treated HMVECs (Figures S5A-B). To examine the contribution of this increased Rac1 activation to the anti-permeability effects of imatinib, we examined endothelial monolayer permeability in HMVECs expressing *Rac1* shRNA (Figure **S5C**). As expected, and in line with the barrier-stabilizing effects of Rac1 activation, *Rac1* knockdown increased baseline permeability in unstimulated cells (Figure **S5D**). However, imatinib inhibited VEGF-induced permeability in HMVECs after *Rac1* knockdown (Figure **S5D**), suggesting that Rac1-independent pathways mediate the endothelial barrier-stabilizing effects of Abl kinase inhibition.

Similar to Rac1, the Rap1 GTPase has been implicated in the regulation of endothelial barrier integrity by promoting cortical actin deposition and VE-cadherin junctional stabilization [48]. Rap1 activation induces maturation of adherens junctions in unstimulated endothelial cells and inhibits thrombin-induced barrier dysfunction [49]. The Abl kinases previously have been demonstrated to regulate Rap1 activation in both T cells and epithelial cells, thereby modulating integrin function [50,51]. Interestingly, Abl kinase inhibition led to increased levels of active Rap1 in unstimulated cells, as well as following VEGF treatment (Figures S6A-B). However, imatinib effectively inhibited VEGF-induced endothelial permeability in cells expressing the negative regulator Rap1 GTPase activating protein (Rap1GAP), which prevented both basal and imatinib-induced Rap1 activation (Figures S6C-D). Thus, neither Rac1 nor Rap1 GTPase activation alone account for the anti-permeability effects of imatinib in endothelial cells.

### Loss of Abl Kinase Activity Impaired Induction of Acto-Myosin Contractility by Endothelial Permeability-Inducing Factors

**Figure 5:**
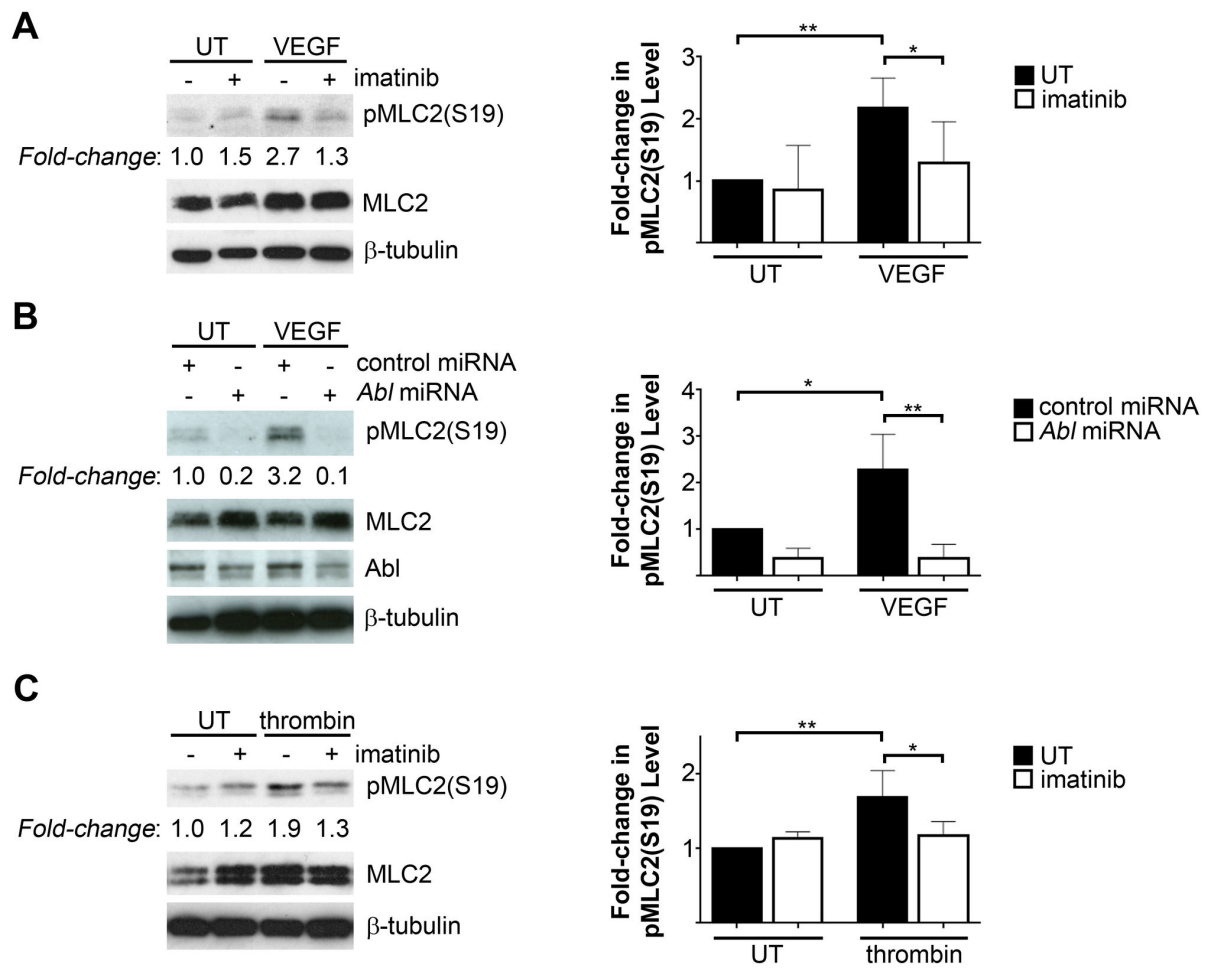
Loss of Abl kinase activity impaired MLC2 phosphorylation in response to endothelial permeability-inducing factors. (**A**) Assessment of phospho-MLC2 serine (S) 19 levels in HMVECs following VEGF stimulation (100ng/mL, 5 minutes) with or without imatinib pre-treatment (10μM). pMLC2(S19) levels, normalized to total MLC2, are quantified in the right panel, relative to levels in untreated cells (UT). Data are presented as means +/- SD (n=7). (**B**) Evaluation of pMLC2(S19) levels in HMVECs expressing either control or Abl miRNAs following VEGF treatment. pMLC2(S19) levels, normalized to total MLC2, are quantified in the right panel, relative to levels in untreated control miRNA-expressing cells (UT). Data are presented as means +/- SD (n=3). (**C**) Assessment of pMLC2(S19) levels in thrombin-treated HMVECs (1U/mL, 2 minutes), with or without imatinib pre-treatment. pMLC2(S19) levels, normalized to total MLC2, are quantified in the right panel, relative to levels in untreated cells (UT). Data are presented as means +/- SD (n=4). *P<0.05; **P<0.01.

In addition to the adhesive forces of cell-cell and cell-matrix interactions, the function of the endothelial barrier is modulated by the generation of contractile forces regulated in part by actin-myosin tension [14,15]. A key determinant of acto-myosin contractility is the phosphorylation of the myosin regulatory light chain (MLC2) at serine (S) 19 or diphosphorylation at threonine 18 and S19, which promotes contractility by increasing myosin ATPase activity [52]. Inhibition of non-muscle myosin II ATPase activity using blebbistatin has previously been shown to decrease thrombin-induced endothelial permeability [53]. Similar to the effects of Abl kinase inhibitors (Figure **4**), treatment of HMVECs with blebbistatin impaired thrombin-induced mislocalization of VE-cadherin (Figure **S7**), suggesting that the generation of acto-myosin contractility is required for disruption of endothelial adherens junctions. Interestingly, while stimulation of HMVECs with VEGF or thrombin increased levels of phospho-MLC2 (S19), the induction of MLC2 phosphorylation by these permeability-inducing factors was decreased by inhibition of Abl kinase activity (Figures 5A,C) or *Abl* knockdown (Figure **5B**). Abl kinase inhibition also impaired formation of actin stress fibers following thrombin stimulation (Figure **S8**). Thus, Abl kinase activity is required for signaling leading to the induction of acto-myosin contractility downstream of endothelial permeability factors.

The phosphorylation status of MLC2 (S19) is regulated by a balance of phosphorylation and dephosphorylation. Phosphorylation of MLC2 in endothelial cells is mediated primarily by the activity of Ca^2+^/calmodulin-dependent myosin light chain kinase (MLCK), as well as the Rho GTPase effector Rho kinase (ROCK), while dephosphorylation is mediated by myosin light chain (MLC) phosphatase [2]. ROCK activity additionally increases levels of MLC2 phosphorylation by phosphorylating and inactivating MLC phosphatase [54]. As loss of Abl kinase activity decreased MLC2 (S19) phosphorylation (Figure **5**), we examined whether Abl kinase activity was required for Rho GTPase activation. We did not detect Rho activation following VEGF or histamine treatment (data not shown). However, thrombin stimulation induced potent activation of Rho GTPase, which was not inhibited in imatinib-treated cells (Figure **S9**), indicating that the decreased MLC2 phosphorylation observed in imatinib-treated cells was not a result of impaired Rho pathway activation.

### Abl Kinase Inhibition Impaired Ca^2+^ Mobilization by Endothelial Permeability-Inducing Factors

**Figure 6:**
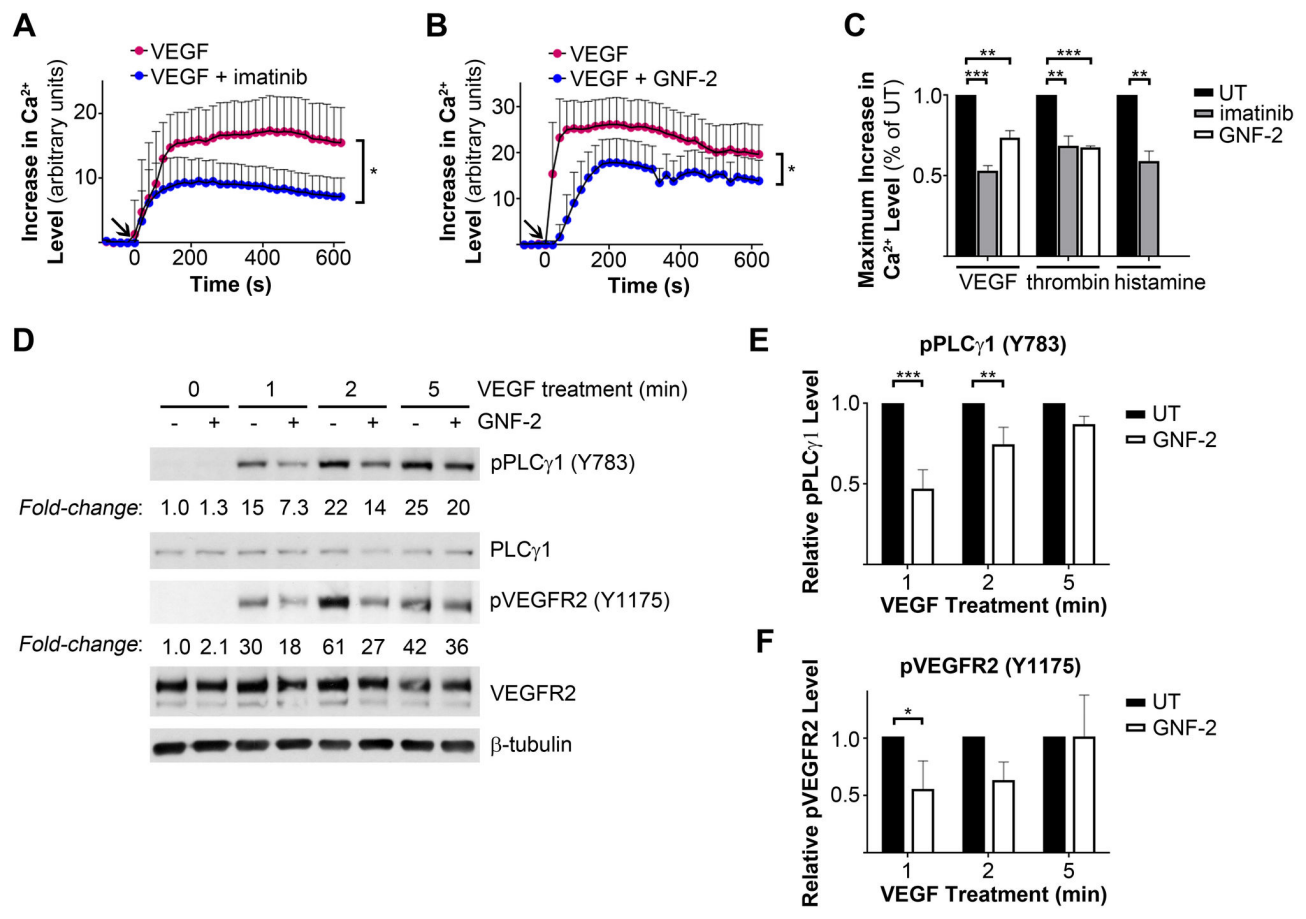
Abl kinase inhibition impaired Ca^2+^ mobilization by endothelial permeability-inducing factors. (**A**-**B**) Quantification of intracellular Ca^2+^ levels in HMVECs stimulated with VEGF (100ng/mL) with or without (**A**) imatinib (10μM) or (**B**) GNF-2 (15μM) pre-treatment. Values are expressed as increases in intracellular Ca^2+^ levels, relative to levels in unstimulated cells. Arrows indicate timing of addition of permeability-inducing factors. Data are presented as means +/- SD of 35 cells per treatment and are representative of 3 independent experiments. (**C**) Quantification of inhibition of Ca^2+^ mobilization by imatinib or GNF-2. Values shown are maximum intracellular Ca^2+^ levels in HMVECs treated with VEGF, thrombin (1U/mL), or histamine (100µM) in the presence of Abl kinase inhibitors, relative to levels in vehicle-treated cells (UT). Data are presented as means +/- SEM (n=3). (**D**) Assessment of VEGF-mediated phosphorylation of PLCγ1 and VEGFR2 in HMVECs, with or without GNF-2 pre-treatment. (**E**-**F**) Quantification of levels of (**E**) phospho-PLCγ1 tyrosine (Y) 783 and (**F**) phospho-VEGFR2 Y1175 in HMVECs treated with VEGF +/- GNF-2, relative to levels in vehicle-treated cells (UT) at each time point. Data are presented as means +/- SD (n=3). *P<0.05; **P<0.01; ***P<0.001.

The lack of inhibition of thrombin-induced Rho GTPase activation by imatinib suggested that Abl kinases regulate acto-myosin contractility through an alternative pathway. Stimulation of endothelial cells with permeability-inducing agonists is known to increase levels of intracellular calcium, mediated both by release of Ca^2+^ from intracellular stores and by extracellular Ca^2+^ entry through plasma membrane channels, contributing to the activation of Ca^2+^/calmodulin-regulated enzymes including MLCK [55,56]. Notably, we observed decreased VEGF-induced Ca^2+^ mobilization in the presence of the Abl kinase inhibitors imatinib or GNF-2 (Figures 6A-B). Increases in intracellular Ca^2+^ levels induced by VEGF, thrombin, or histamine were decreased by 30-50% following Abl kinase inhibition (Figure **6C**). The release of Ca^2+^ from intracellular stores is triggered by the binding of inositol-1,4,5-trisphosphate (IP_3_) to its cognate receptor on the endoplasmic reticulum (ER) membrane. Cellular IP_3_ levels are, in turn, regulated by the activity of phosphoinositide-specific phospholipase C (PLC) family enzymes. In endothelial cells, VEGF-mediated IP_3_ generation is regulated by PLCγ activation downstream of VEGF receptor 2 (VEGFR2) [57]. We observed delayed PLCγ1 activation following VEGF stimulation in HMVECs treated with the Abl kinase inhibitor GNF-2, as assessed by levels of activating phosphorylation of PLCγ1 (Y783) [58] (Figures 6D-E). The levels of phosphorylated PLCγ1 (Y783) induced by VEGF stimulation were reduced by 50% in GNF-2-treated cells 1 minute after VEGF treatment (Figure **6E**). The effect of GNF-2 was transient, as comparable levels of phospho-PLCγ1 (Y783) were detected in vehicle- and GNF-2-treated cells by 5 minutes post-VEGF stimulation. Similar findings were observed upon VEGF stimulation in the presence of imatinib (data not shown). VEGF-mediated PLCγ1 activation requires phosphorylation of VEGFR2 on Y1175 [59]. Interestingly, Abl kinase inhibition also impaired VEGF-induced phosphorylation of VEGFR2 (Y1175) at earlier time points (1 to 2 minutes) (Figures 6D,F). Taken together, these findings suggest that Abl kinase inhibition impairs the VEGF-induced mobilization of intracellular Ca^2+^ in part through decreased VEGF receptor phosphorylation and resulting inhibition of PLCγ activation. 

## Discussion

**Figure 7:**
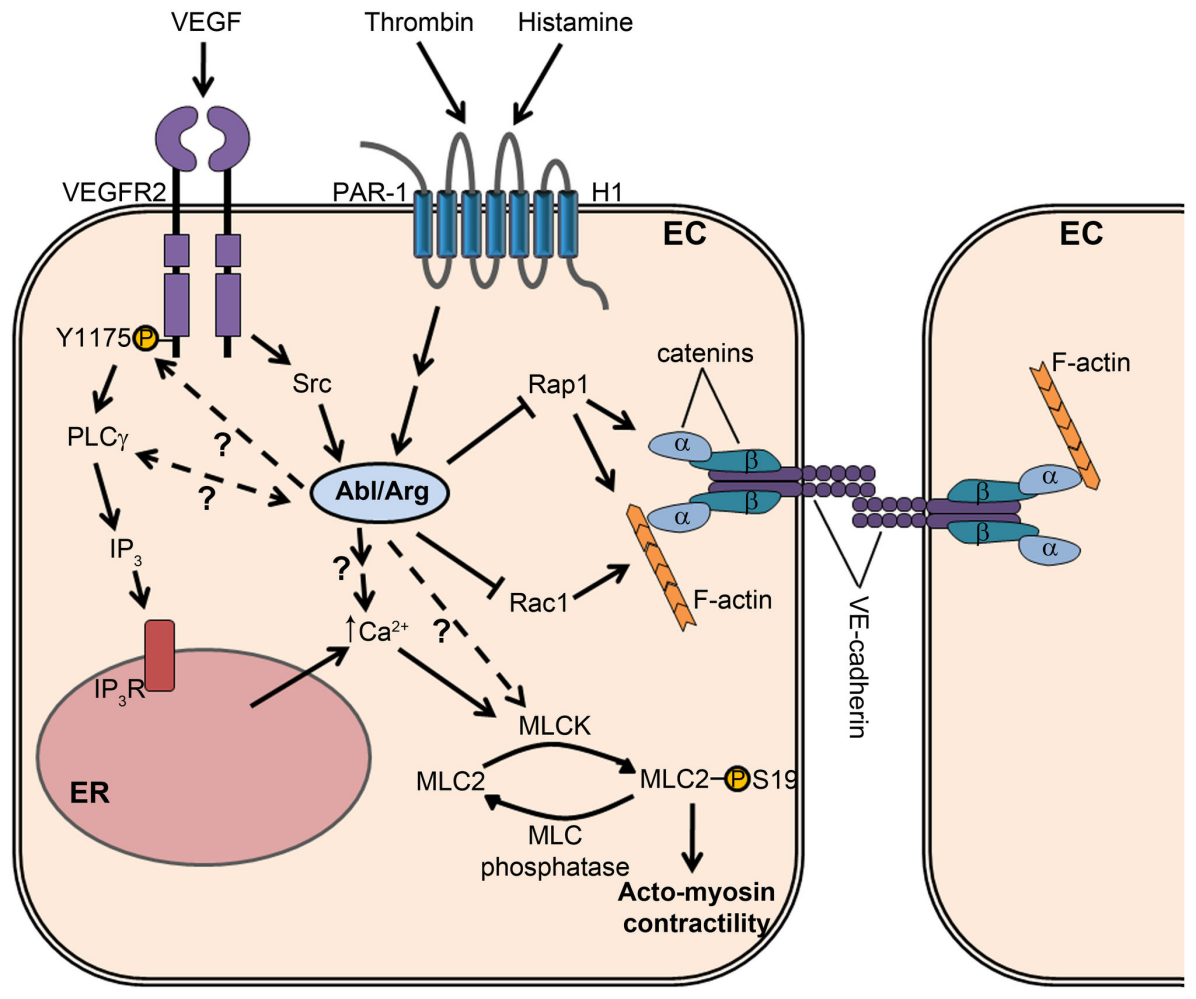
Model for the role of the Abl family kinases in signaling pathways regulating endothelial permeability. The Abl and Arg kinases are activated in endothelial cells downstream of receptors for the permeability-inducing factors VEGF, thrombin, and histamine. VEGF-mediated Abl kinase activation requires Src family kinase activity. The Abl kinases positively regulate phosphorylation of MLC2 (S19) in response to these permeability-inducing agonists, likely through regulating the activity of Ca^2+^/calmodulin-dependent targets such as MLCK. Abl kinase activity is required for maximal Ca^2+^ mobilization in response to stimulation with permeability-inducing factors. The Abl kinases additionally modulate VEGF-induced phosphorylation of VEGFR2 at Y1175, which regulates downstream PLCγ activation, IP_3_ generation, and ER Ca^2+^ release. Abl kinases promote Ca^2+^ mobilization by thrombin and histamine by mechanisms yet to be characterized. Abl kinases negatively regulate basal activity levels of the Rac1 and Rap1 GTPases, which have been shown to support endothelial barrier function by promoting cortical actin deposition and adherens junction stability. Abbreviations: EC, endothelial cell; VEGF, vascular endothelial growth factor; VEGFR2, VEGF receptor 2; Y, tyrosine; PAR-1, protease-activated receptor 1 (thrombin); H1, histamine H1 receptor; MLC2, myosin regulatory light chain; S, serine; MLCK, myosin light chain kinase; PLC, phospholipase C; IP_3_, inositol-1,4,5-trisphosphate; IP_3_R, IP_3_ receptor; ER, endoplasmic reticulum.

Excessive vascular leakage is a feature of a wide range of pathological conditions, leading to complications including edema and increased tissue damage following ischemic stroke and myocardial infarction, increased interstitial fluid pressure in cancers, and pulmonary dysfunction in acute respiratory distress syndrome [3,4]. Efforts to prevent this increased vascular permeability are complicated, in part, by the multiple permeability-inducing factors involved in these disorders [60]. In the current study, we have identified the Abl family kinases, Abl and Arg, as mediators of endothelial barrier dysfunction induced by several disparate permeability factors, including agonists signaling through both receptor tyrosine kinases (VEGF) and G protein-coupled receptors (thrombin and histamine). Importantly, Abl kinase inhibition, using either imatinib or the Abl/Arg-specific allosteric inhibitor GNF-2, impaired VEGF-induced vascular permeability both in cultured endothelial cells and in mice. We showed for the first time a direct requirement for Abl in VEGF-induced dermal vascular leakage in mice lacking endothelial *Abl* expression. Loss of Abl kinase activity protects against endothelial barrier dysfunction; this effect is accompanied by activation of the barrier-stabilizing GTPases Rac1 and Rap1, as well as inhibition of agonist-induced Ca^2+^ mobilization and generation of acto-myosin contractility. A model for the proposed role of the Abl kinases in signaling pathways regulating endothelial permeability is shown in Figure **7**.

The endothelial barrier is regulated by a dynamic balance of adhesive and contractile forces [2]. We found that inhibition of the Abl kinases led to increased activation of the endothelial barrier-promoting GTPases Rac1 and Rap1, which promote cortical actin remodeling and adherens junction stability [45–49]. The Abl kinases previously have been linked either to positive or negative regulation of GTPase activation, depending upon the cellular context. The Abl kinases are required for Rac1 activation downstream of cadherin engagement in epithelial cells [21], as well as Rap1 activation following T cell receptor engagement or chemokine stimulation in T cells [51,61]. In contrast, expression of constitutively-active Abl kinases reduced levels of active Rap1 in epithelial and HEK293 cells, as a result of Abl-mediated tyrosine phosphorylation of the CrkII adaptor and disruption of the association between CrkII and the Rap1 guanine nucleotide exchange factor (GEF) C3G [50,62]. It remains to be determined whether the enhanced Rap1 activation we observed upon Abl kinase inhibition in endothelial cells similarly results from increased CrkII/C3G interaction and C3G GEF activity. However, our findings that reduced Rac1 expression or Rap1 activity did not prevent imatinib-mediated endothelial barrier stabilization suggest that Rac1- and Rap1-independent pathways mediate the anti-permeability effects of Abl/Arg kinase inhibition.

Notably, inhibition of Abl kinase activity impaired VEGF- and thrombin-induced phosphorylation of the myosin regulatory light chain (MLC2), which regulates myosin ATPase activity and induction of barrier-destabilizing acto-myosin contractility [52], and inhibited thrombin-induced formation of actin stress fibers. However, Abl kinase inhibition did not affect activation of the Rho/ROCK pathway in endothelial cells, which increases MLC2 phosphorylation through inhibitory phosphorylation of myosin light chain phosphatase [54]. In contrast, in fibroblasts, the Arg kinase previously has been linked to inhibition of acto-myosin contractility during integrin-mediated adhesion and migration, through phosphorylation and activation of the RhoA inhibitor p190RhoGAP [63]. Similarly, Abl kinase inhibition in epithelial cells led to increased baseline Rho activation, formation of actin stress fibers, and weakened intercellular adhesion [21]. These disparate findings may be explained by differential roles for Abl kinases in the regulation of signaling pathways mediating basal versus agonist-induced Rho activation and acto-myosin contractility in various cell types.

Phosphorylation of the myosin regulatory light chain can be regulated by the Ca^2+^/calmodulin-dependent myosin light chain kinase (MLCK) [55]. Interestingly, treatment with the endothelial barrier-promoting bioactive lipid sphingosine-1-phosphate was shown to increase Abl-mediated MLCK (Y464) phosphorylation [29], raising the possibility that Abl kinase activation by endothelial permeability-inducing mediators similarly may contribute to MLCK phosphorylation and activation. Tyrosine phosphorylation of MLCK has been linked to enhanced enzymatic activity at lower Ca^2+^ concentrations [64]. Notably, we found that increases in intracellular Ca^2+^ levels induced by endothelial barrier-disrupting factors were attenuated by Abl kinase inhibition. Thus, we postulate that decreased intracellular Ca^2+^ mobilization in cells with reduced Abl kinase activity might contribute to impaired agonist-stimulated MLCK activity, leading to the observed reduction in MLC2 phosphorylation. Further investigation is needed to fully characterize the effects of Abl kinase inhibition on MLCK activity, as well as to determine whether the impaired Ca^2+^ mobilization inhibits activation of other Ca^2+^-regulated enzymes involved in endothelial barrier dysfunction, such as PKCα [57]. 

Consistent with the observed impairment of agonist-mediated Ca^2+^ mobilization, we found that VEGF-induced PLCγ activation was delayed in the absence of Abl kinase activity. Interestingly, phosphorylation of VEGFR2 (Y1175), which is required for PLCγ activation [59], similarly was delayed in endothelial cells treated with Abl kinase inhibitors. Previous work has demonstrated Abl kinase-mediated phosphorylation of the PDGF and epidermal growth factor receptors [65,66]; however, it remains to be determined whether VEGFR2 is an Abl kinase target. In addition, we have previously identified a bi-directional link between PLCγ and Abl in PDGF-stimulated fibroblasts, whereby PLCγ is required for Abl kinase activation, and in turn, Abl modulates PLCγ enzymatic activity [67]. Further investigation will be required to determine if a similar PLCγ-Abl connection exists in VEGF-stimulated endothelial cells. In all, these findings suggest that impaired VEGF-induced Ca^2+^ mobilization upon Abl kinase inhibition may result, at least in part, from decreased PLCγ-mediated IP_3_ generation, resulting in impaired release of endoplasmic reticulum (ER) Ca^2+^ stores. However, distinct mechanisms are likely to be involved in the inhibition of thrombin- and histamine-induced Ca^2+^ mobilization, which is regulated by G protein-mediated activation of PLCβ [57]. In this regard, previous studies have suggested a role for tyrosine kinases in regulating extracellular calcium entry through plasma membrane channels following depletion of intracellular Ca^2+^ stores (store-operated Ca^2+^ influx) [57].

In summary, we have demonstrated a requirement for the Abl kinases in induction of endothelial permeability by VEGF and the inflammatory mediators thrombin and histamine. Our findings and recent reports suggest that the endothelial barrier-protective effects of Abl kinase inhibition result from several distinct mechanisms, including promoting cell-cell and cell-matrix adhesion [28], as well as impairing induction of acto-myosin contractility. While the precise Abl kinase targets involved in the regulation of these pathways remain to be characterized, the existence of multiple pathways mediating the anti-permeability effects of Abl kinase inhibition suggests that pharmacological targeting of the Abl kinases may be capable of inhibiting endothelial permeability induced by a broad range of agonists. However, further studies will be needed to evaluate the involvement of the Abl kinases in endothelial barrier dysfunction mediated by additional permeability-inducing factors, as well as to determine whether *in vivo* pharmacological or genetic inactivation of the Abl kinases may have protective effects in disorders involving pathological vascular leakage.

## Materials and Methods

### Mice and Ethics Statement

The generation of endothelial *Abl* knockout mice was described previously [31]. Mice were housed under specific pathogen-free conditions in the Duke University Cancer Center Isolation Facility. All animal procedures used in this study were reviewed and approved by the Duke University Institutional Animal Care and Use Committee (protocols A183-07-07, A152-10-06, and A137-13-05) and were carried out in accordance with the Guide for the Care and Use of Laboratory Animals (National Research Council). 

### Inhibitors and Reagents

Imatinib (Gleevec/STI571) was a generous gift from Novartis and purchased from LGM Pharma. GNF-2 and su6656 were purchased from Sigma. Recombinant human vascular endothelial growth factor (VEGF-A-165) was purchased from R&D Systems. Thrombin from human plasma and histamine were purchased from Sigma. N_ω_-Nitro-L-arginine methyl ester hydrochloride (L-NAME) and (-)-blebbistatin were purchased from Sigma. S-Nitroso-*N*-Acetylpenicillamine (SNAP) was purchased from Life Technologies.

### Cell Culture

Human microvascular endothelial cells (HMVECs) immortalized with telomerase reverse transcriptase (hTERT) were provided by Xiao-Fan Wang (Duke University Medical Center) [68]. Endothelial cells routinely were cultured in microvascular endothelial growth medium-2 (EGM-2MV; Lonza). For experiments examining signaling responses to permeability-inducing factors, confluent HMVECs were serum-starved approximately 16 hours in endothelial basal medium-2 (EBM-2) supplemented with 0.2% (wt/vol) bovine serum albumin (BSA). Cells were pre-treated with the Abl kinase inhibitors imatinib (10μM) or GNF-2 (15μM), the Src kinase inhibitor su6656 (1μM), or the eNOS inhibitor L-NAME (200µM) for one hour prior to treatment with VEGF (100ng/mL), thrombin (1U/mL), or histamine (100μM).

### Viral Transduction

Control (non-specific) and human *Rac1* shRNA oligos were cloned into pSuper-Retro-puro (pSR-puro) retroviral vector (OligoEngine). shRNA sequences (antisense) were as follows: control shRNA – AAA TGT ACT GCG CGT GGA G; *Rac1* shRNA – TTT TAC AGC ACC AAT CTC C. pSR-puro shRNA constructs were transfected into 293T cells, along with pCL10A-1 packaging vector, using FuGENE6 reagent (Promega). Retroviral supernatants were collected and filtered 48 hours post-transfection. HMVECs were incubated 16 to 24 hours with retroviral medium in the presence of 6μg/mL Polybrene. Transduction with pBabe-puro retroviral constructs (pBabe-puro vector; pBabe-puro/mAbl-WT; pBabe-puro/hRap1GAP) was performed similarly. Transduced cells were selected in 1μg/mL puromycin for at least two days. Lentiviral micro-RNA (miRNA)-mediated knockdown of *Abl*/*Arg* in HMVECs was conducted as described previously [50,69]. miRNA targeting sequences were as follows: control miRNA – GGT GTA TGG GCT ACT ATA GAA; *Abl*/*Arg* dual miRNA – *Abl* miRNA – GGT GTA TGA GCT GCT AGA GAA and *Arg* miRNA – AGG TAC TAA AGT GGC TCT GAG. *Abl* single knockdown was performed using a single lentiviral miRNA vector expressing 3 *Abl* miRNAs in tandem (miRNA Abl 6/2/1; generously provided by Owen Witte, University of California, Los Angeles, [70]). HMVECs were plated for signaling experiments and Transwell permeability assays 48 hours after miRNA viral transduction.

### Antibodies

Antibodies used for Western blotting included phospho-CrkL (Y207), phospho-PLCγ1 (Y783), phospho-VEGFR2 (Y1175), VEGFR2, phospho-MLC2 (S19), MLC2, phospho-Src (Y416), phospho-eNOS (S1177), phospho-paxillin (Y118), phospho-FAK (Y576/577), and Rap1 from Cell Signaling; β-tubulin from Sigma-Aldrich; CrkL (C-20), Src (SRC 2), Arg (9H5), PLCγ1 (530), FAK (C-20-G), and Rap1GAP (H-93) from Santa Cruz; Abl (8E9), paxillin, and Rac1 from BD Biosciences; eNOS from Bethyl Laboratories; Rho and Rac1 from Pierce (Thermo Scientific); and mouse anti-α-catenin from Zymed (Life Technologies). Antibodies used for both Western blotting and immunoprecipitation included VE-cadherin (C-19) from Santa Cruz and β-catenin from BD Biosciences. VE-cadherin antibody (anti-Cadherin-5) from BD Biosciences was used for immunofluorescence.

### Lysis and Western Blotting

Cells were washed once with ice-cold phosphate-buffered saline (PBS), then lysed in radioimmunoprecipitation assay (RIPA) buffer [50mM Tris-HCl, pH 7.5; 150mM NaCl; 1% Triton X-100; 0.1% sodium dodecyl sulfate (SDS); 1% sodium deoxycholate; 0.05% NP-40; 5mM ethylenediaminetetraacetic acid (EDTA)] with protease/phosphatase inhibitors [0.1mM phenylmethane sulfonyl fluoride (PMSF); 1μg/mL aprotinin; 1μg/mL leupeptin; 10μg/mL pepstatin; 10mM β-glycerophosphate; 1mM sodium fluoride; 0.1mM sodium orthovanadate]. Cell debris was removed by microcentrifugation, and protein concentrations were quantified using Bio-Rad *DC* protein assay reagents. Equal amounts of protein were separated by SDS-polyacrylamide gel electrophoresis (SDS-PAGE) and transferred to nitrocellulose membranes. Membranes were incubated with primary antibodies in blocking solution containing either 5% (wt/vol) non-fat dry milk or 3% (wt/vol) BSA in Tris-buffered saline-Tween 20 (TBS-T), overnight at 4°C. Blots were washed three times with TBS-T, then incubated with horseradish peroxidase-coupled secondary antibodies (Jackson ImmunoResearch or Santa Cruz) in blocking solution for one hour at room temperature. Blots were washed with TBS-T and developed using enhanced chemiluminescence (ECL) Western blotting detection reagent (Amersham/GE Healthcare). Western blots were quantified using ImageJ analysis software (NIH).

### Biotinylation of Cell Surface Protein

Confluent HMVECs were serum-starved overnight (EBM-2 with 0.2% BSA), pre-treated for 1 hour with imatinib (10μM), then stimulated for 15 or 30 minutes with VEGF (100ng/mL) at 37°C. Cells were immediately moved to 4°C for the remainder of the procedure. Cell surface proteins were biotinylated with EZ-link Sulfo-NHS-SS-biotin (Thermo Scientific; 0.4mg/mL in PBS, with calcium and magnesium) for 30 min, followed by PBS washes and quenching of unreacted biotin in 50mM ammonium chloride in PBS with calcium and magnesium (2x5 minute washes). Following PBS washes, cells were lysed in RIPA Buffer (with protease/phosphatase inhibitors). For isolation of biotinylated cell surface proteins, equal amounts of each lysate (250μg) were incubated with high-capacity NeutrAvidin Agarose resin (Thermo Scientific) for 1 hour at 4°C with constant mixing. Beads were washed four times with PBS with 1% NP-40 and protease/phosphatase inhibitors, then boiled in 2X reducing SDS-PAGE sample buffer for analysis by western blotting.

### Immunoprecipitation

Confluent HMVECs were serum-starved overnight (EBM-2 with 0.2% BSA), pre-treated for 1 hour with imatinib (10μM), then stimulated for 5 or 15 minutes with VEGF (100ng/mL). Cells were washed once with ice-cold PBS, then lysed in NP-40 lysis buffer (50mM Tris-HCl, pH 8.0, with 150mM sodium chloride and 1% NP-40) with protease/phosphatase inhibitors. Equal amounts of each lysate (300μg, pre-cleared) were incubated with either VE-cadherin (0.6μg) or β-catenin (0.5μg) antibodies overnight at 4°C with constant mixing, followed by incubation with Protein G Sepharose 4 Fast Flow beads (GE Healthcare Life Sciences) for 6 hours at 4°C. Beads were washed four times with NP40 buffer with protease/phosphatase inhibitors, then boiled in 2X reducing SDS-PAGE sample buffer for analysis by western blotting.

### GTPase Activation Assays

Confluent HMVECs were serum-starved overnight (EBM-2 with 0.2% BSA), pre-treated with 10μM imatinib for 1 hour, then stimulated for 2 minutes with VEGF (100ng/mL) or thrombin (1U/mL). Activity of Rac1, Rap1, and Rho GTPases was examined using Active GTPase Pull-down and Detection kits (Pierce/Thermo Scientific), following the manufacturer’s protocol.

### Immunofluorescence

HMVECs were cultured to confluence on glass coverslips, then serum-starved approximately 16 hours in EBM-2 basal medium with 0.2% BSA. Cells were pre-treated with either imatinib (10μM), GNF-2 (15μM), or blebbistatin (5μM) one hour prior to treatment with VEGF (100ng/mL, 30 minutes) or thrombin (1U/mL, 5 minutes). Cells were washed with ice-cold PBS, then fixed 15 minutes in 4% paraformaldehyde (in PBS) and permeabilized 15 minutes in 3% BSA in PBS with 0.05% sodium azide and 0.05% Triton X-100 (VE-cadherin staining) or 0.1% Triton X-100 (phalloidin staining). VE-cadherin primary antibody (anti-Cadherin-5; BD Biosciences) was added to coverslips at 1:200 dilution in blocking buffer (3% BSA in PBS with 0.05% sodium azide) for one hour at room temperature. Following PBS washes, Alexa 568- or Alexa 488-coupled goat anti-mouse IgG (Life Technologies) was added at 1:250 dilution in blocking buffer for one hour at room temperature, followed by DNA counterstaining with Hoechst 33342 (0.5μg/mL, 5 minutes). For staining of the actin cytoskeleton, Alexa 488-conjugated phalloidin (Life Technologies) was added at 1:100 dilution in blocking buffer for one hour at room temperature, followed by DNA counterstaining. Images were acquired using a Zeiss Axiovert 200M fluorescence microscope and AxioVision software (Rel. 4.8).

### Transwell Permeability Assay

Transwell supports (6.5mm diameter, 0.4μm pore size, polyester membranes; Corning) in 24-well tissue-culture plates were pre-coated with 0.02mg/mL bovine fibronectin (Sigma) for one hour at 37°C, prior to seeding of HMVECs at 2.7x10^4^ cells per Transwell. HMVECs were cultured in Transwells for three days at 37°C to allow formation of a confluent monolayer. Cells were then pre-treated with Abl kinase inhibitors in serum-free medium (EBM-2 with 0.2% BSA) for one hour prior to permeability assay. Fluorescein-labeled dextran (anionic, molecular weight 40kDa; Invitrogen/Life Technologies) was added to the top chamber of each Transwell at 1mg/mL, followed by treatment with permeability-inducing factors. Each treatment was performed in triplicate wells. At the indicated times, 50μL samples were removed from the bottom chamber of each Transwell and replaced with 50μL fresh culture medium. The collected samples were diluted 20-fold with PBS and fluorescence measured (excitation 485nm/emission 535nm) using a VICTOR^3^ plate reader (PerkinElmer) and Wallac 1420 Workstation software.

### In Vivo Permeability Assays (Modified Miles Assay)

#### Abl Kinase Inhibition

*In vivo* vascular permeability was examined using a modified Miles assay [71], examining leakage of Evans blue dye as an indicator of albumin extravasation. Approximately 12-week-old female athymic nude mice were anesthetized with 100mg/kg ketamine and 10mg/kg xylazine. Evans blue dye (Sigma; 30mg/kg in 100μL sterile saline) was administered by tail vein injection, followed by intradermal injections of PBS and VEGF (100ng in 30μL PBS) into the back skin, co-injected with vehicle or either of two Abl kinase inhibitors (imatinib or GNF-2, 15 μM). Fifteen minutes after intradermal injections, mice were humanely euthanized by CO_2_ inhalation and the back skin surrounding each injection site dissected, blotted dry, and weighed. Evans blue dye was extracted from skin tissue samples in formamide (Sigma) overnight at 56°C, and absorbance measured at 620nm. Amounts of extracted Evans blue dye were calculated based on a standard curve of Evans blue dye in formamide and normalized to skin tissue sample weight.

#### Endothelial Abl Knockout Mice

The modified Miles assay described above was also performed using approximately 16-week-old endothelial *Abl* knockout mice (*Abl* ^*flox/flox*^ *; Arg* ^*+/-*^ *; Tie2-Cre* ^*+/-*^, referred to as *Abl* ^*ECKO*^ *; Arg* ^*+/-*^) and age- and sex-matched *Arg* ^*+/-*^ controls (*Abl* ^*flox/flox*^ *; Arg* ^*+/-*^ *; Tie2-Cre* ^*-/-*^). Mice were anesthetized with 100mg/kg ketamine and 10mg/kg xylazine, followed by intravenous administration of 30mg/kg Evans blue dye. Back hair was shaved prior to intradermal injections of PBS and VEGF (100ng in 30μL PBS). Mice were euthanized 15 minutes after VEGF administration, and Evans blue dye was extracted and quantified as described above.

### Analysis of Intracellular Ca^2+^ Levels

HMVECs were cultured to confluence on 35mm glass bottom microwell dishes (MatTek Corporation; Ashland, MA, USA), then serum-starved overnight in EBM-2 basal medium with 0.2% BSA. Cells were loaded with 3μM cell-permeant Calcium Green-1, AM fluorescent Ca^2+^ indicator (Molecular Probes/Life Technologies) in the presence of 0.1% Pluronic F-127 in serum-free medium for 45 minutes at room temperature. Cells were washed three times with Hank’s Balanced Salt Solution (HBSS; with calcium and magnesium, without phenol red), then pre-treated with imatinib (10μM) or GNF-2 (15μM) in HBSS for 1 hour. Intracellular Ca^2+^ levels, as assessed by Calcium Green-1 fluorescence, were measured by live cell imaging using a Zeiss Axio Observer Z1 fluorescence microscope (GFP filtercube: BP 470/40, FT 495, BP 525/50) and MetaMorph software. Images were acquired using four microscope fields per treatment. Three images were acquired prior to addition of permeability-inducing factors, for assessment of baseline intracellular Ca^2+^ levels. Images were acquired for 10 minutes (at 20 second intervals) following addition of VEGF (100ng/mL), thrombin (1U/mL), or histamine (100μM) in HBSS. Average fluorescence intensity (with baseline subtracted) was measured on a single-cell basis for 35 cells per treatment, using Metamorph software.

### Analysis of Nitric Oxide Production

HMVECs were cultured to confluence on 35mm glass bottom microwell dishes (MatTek Corporation; Ashland, MA, USA), then serum-starved overnight in EBM-2 basal medium with 0.2% BSA. Cells were loaded with 3μM DAF-FM diacetate fluorescent nitric oxide indicator (Life Technologies) in serum-free medium for 45 minutes at 4°C. Cells were washed three times with HBSS (with calcium and magnesium, without phenol red), then pre-treated with imatinib (10μM) in HBSS for 1 hour. Nitric oxide (NO) levels, as assessed by DAF-FM fluorescence, were measured by live cell imaging using a Zeiss Axio Observer Z1 fluorescence microscope (GFP filtercube: BP 470/40, FT 495, BP 525/50) and MetaMorph software. 3 images were acquired prior to addition of VEGF for assessment of baseline NO levels, and images were acquired for 1 hour (at 10 minute intervals) following treatment with 100ng/mL VEGF (in HBSS). Average fluorescence intensity (with baseline subtracted) was measured for four (20X) microscope fields per treatment, using MetaMorph software.

### Statistical Analysis

All statistical analyses were performed using GraphPad Prism 6 software. Comparisons of two groups were performed using Student *t* tests (two-tailed). Comparisons involving multiple groups were evaluated using one-way ANOVA, followed by Bonferroni posttests. Two-way ANOVA, followed by Bonferroni posttests, was used to evaluate changes in HMVEC permeability in multiple treatment groups over time. For all tests, P<0.05 was considered statistically significant.

## Supporting Information

Figure S1 **Abl kinase inhibition did not alter VEGF-induced Src activation.** (**A**) Assessment of phospho-Src (Y416) levels in HMVECs treated for the indicated times with 100ng/mL VEGF +/- 10μM imatinib. Phospho-Src (Y416) levels, normalized to total Src levels, are quantified in the bottom panel. Values are presented as means +/- SD (n=3), relative to levels in VEGF-treated cells (5 min). *P<0.05; ns = not significant. (**B**) Evaluation of VEGF-induced phosphorylation of paxillin (Y118) and FAK (Y576/577) in HMVECs pre-treated with 10μM imatinib, 1μM su6656, or vehicle control (UT).
(TIF)

Figure S2 **Impaired endothelial permeability following *Abl* knockdown.** (**A** and **B**) Assessment of permeability of HMVEC monolayers expressing either control or *Abl* miRNAs to fluorescein-labeled dextran (molecular weight 40kDa), following (**A**) VEGF (100ng/mL) or (**B**) thrombin (1U/mL) treatment. Data shown are mean fluorescence of samples collected from bottom Transwell chambers at the indicated times following VEGF or thrombin treatment, +/- SD of three replicates per treatment. Data are representative of 4-5 independent experiments. (**C**) Quantification of inhibition of VEGF- and thrombin-induced endothelial permeability following *Abl* knockdown. Values are expressed relative to permeability of HMVECs expressing control miRNA. Data are presented as means +/- SEM (VEGF, n=5; thrombin, n=4). (**D**) Evaluation of baseline permeability to fluorescein-labeled dextran of unstimulated HMVEC monolayers expressing control, *Abl*, or *Abl*/*Arg* miRNAs. Data are presented as means +/- SEM (n=4). (**E**) Assessment of Abl and Arg protein levels in HMVECs following *Abl* or *Abl*/*Arg* knockdown. *P<0.05; **P<0.01; ***P<0.001.
(TIF)

Figure S3 **Abl kinase inhibition did not alter VE-cadherin cell surface levels or adherens junction complex association.** (**A**) Evaluation of total and cell surface VE-cadherin protein levels in HMVECs treated with VEGF (100ng/mL) with or without imatinib pre-treatment (10μM), as assessed by biotinylation of cell surface proteins. Cell surface VE-cadherin levels are quantified in the right panel, relative to levels in untreated cells (UT). Data are presented as means +/- SD (n=3). (**B**) Assessment of VE-cadherin association with β-catenin in HMVECs treated with VEGF +/- imatinib, following VE-cadherin immunoprecipitation. Data are quantified in the right panel as means +/- SD (n=5), relative to co-immunoprecipitated β-catenin levels in vehicle-treated cells (UT) at each time point. (**C**-**E**) Assessment of β-catenin association with VE-cadherin and α-catenin in HMVECs treated with VEGF +/- imatinib, following β-catenin immunoprecipitation. (**D**-**E**) Quantification of levels of co-immunoprecipitated (**D**) VE-cadherin and (**E**) α-catenin, relative to levels in vehicle-treated cells (UT) at each time point. Data are presented as means +/- SD (VE-cadherin, n=5; α-catenin, n=2).
(TIF)

Figure S4 **No effect of Abl kinase inhibition on VEGF-induced nitric oxide production.** (**A**) Assessment of eNOS (S1177) phosphorylation in HMVECs following 5 or 15 minutes treatment with 100ng/mL VEGF, in the absence (UT) or presence of 10μM imatinib. Phospho-eNOS (S1177) levels, normalized to total levels, are quantified in the right panel. Values are expressed as means +/- SD (n=3), relative to levels in VEGF-treated cells (5 min). (**B**) Evaluation of VEGF-induced nitric oxide (NO) production in HMVECs, +/- imatinib, relative to levels in unstimulated cells. Values are expressed as means +/- SD of 4 fields per treatment and are representative of 3 independent experiments. (**C**) Evaluation of endothelial monolayer permeability, as assessed by passage of fluorescein-labeled dextran (molecular weight 40kDa) through HMVEC monolayers grown on Transwells, following treatment with VEGF (100ng/mL, 60 minutes) with or without imatinib pre-treatment, in the absence (UT) or presence of the NO donor SNAP (100μM). Data shown are mean fluorescence of samples collected from bottom Transwell chambers, +/- SD of three replicates per treatment. Data are representative of three independent experiments. (**D**) Assessment of Abl kinase activation, as determined by phospho-CrkL tyrosine (Y) 207 levels, following stimulation of serum-starved HMVECs with 100ng/mL VEGF for 5 or 15 minutes, with or without pre-treatment with 10μM imatinib or 200μM L-NAME. pCrkL (Y207) levels (normalized to total CrkL) are quantified in the right panel, relative to levels in untreated (UT) cells. Data are presented as means +/- SD (n=3). *P<0.05; **P<0.01; ***P<0.001; ns = not significant.
(TIF)

Figure S5 **Increased Rac1 GTPase activity following Abl kinase inhibition.** (**A**-**B**) Assessment of levels of GTP-bound (active) Rac1 GTPase in HMVECs treated with imatinib (10μM), then treated with VEGF (100ng/mL, 2 minutes) or left unstimulated (UT). Rac1-GTP levels, normalized to total Rac1, are quantified in (**B**), relative to levels in vehicle-treated cells (UT). Data are presented as means +/- SD (n=2). (**C**) Assessment of Rac1 protein levels following *Rac1* shRNA expression. (**D**) Evaluation of permeability of HMVECs expressing either control or *Rac1* shRNAs to fluorescein-labeled dextran, following 60 minutes VEGF stimulation with or without imatinib pre-treatment. Data shown are mean fluorescence of samples collected from bottom Transwell chambers, +/- SD of three replicates per treatment. Data are representative of three independent experiments. *P<0.05; **P<0.01; ***P<0.001.
(TIF)

Figure S6 **Increased Rap1 GTPase activity following Abl kinase inhibition.** (**A**-**B**) Assessment of levels of GTP-bound (active) Rap1 GTPase in HMVECs treated with imatinib (10μM), either treated with VEGF (100ng/mL, 2 minutes) or left unstimulated (UT). Rap1-GTP levels, normalized to total Rap1, are quantified in (**B**), relative to levels in vehicle-treated cells (UT). Data are presented as means +/- SD (n=5). (**C**) Evaluation of permeability of HMVECs expressing either Rap1GAP or vector control to fluorescein-labeled dextran, following 60 minutes VEGF treatment with or without imatinib pre-treatment. Data shown are mean fluorescence of samples collected from bottom Transwell chambers, +/- SD of three replicates per treatment. Data are representative of two independent experiments. (**D**) Assessment of levels of active, GTP-bound Rap1 in vehicle (UT)- or imatinib-treated cells expressing either Rap1GAP or vector control. *P<0.05; **P<0.01; ***P<0.001.
(TIF)

Figure S7 **Inhibition of acto-myosin contractility impaired thrombin-induced disruption of endothelial adherens junctions.** Staining of HMVEC monolayers for the adherens junction marker VE-cadherin (red) following treatment with thrombin (1U/mL, 5 minutes), with or without pre-treatment with the non-muscle myosin II ATPase inhibitor blebbistatin (5μM). Thrombin treatment induced formation of inter-endothelial cell gaps (arrows) and destabilization of endothelial cell-cell junctions (“zig-zag” VE-cadherin staining pattern, arrowheads), which were reduced by blebbistatin pre-treatment.
(TIF)

Figure S8 **Abl kinase inhibition impaired thrombin-induced formation of actin stress fibers.** Evaluation of actin cytoskeletal structure, as assessed by phalloidin staining, in HMVECs treated with thrombin (1U/mL, 5 minutes), either in the absence (UT) or presence of 10μM imatinib. Thrombin treatment resulted in formation of actin stress fibers (arrows) and intercellular gaps (arrowheads), which were inhibited by imatinib pre-treatment.
(TIF)

Figure S9 **Abl kinase inhibition did not affect thrombin-induced activation of Rho GTPase.** Assessment of levels of GTP-bound (active) Rho GTPase in HMVECs either left unstimulated (UT) or treated with thrombin (1U/mL, 2 minutes), +/- imatinib (10μM). Rho-GTP levels, normalized to total Rho, are quantified in the right panel, relative to levels in thrombin-stimulated cells. Data are presented as means +/- SD (n=5). ***P<0.001.
(TIF)
