# Supplementary figures and images for: Abl Family Kinases Regulate Endothelial Barrier Function In Vitro and in Mice
Source: PLoS One. 2013 Dec 19;8(12):e85231. doi: 10.1371/journal.pone.0085231 (PMC3868616; doi:10.1371/journal.pone.0085231)

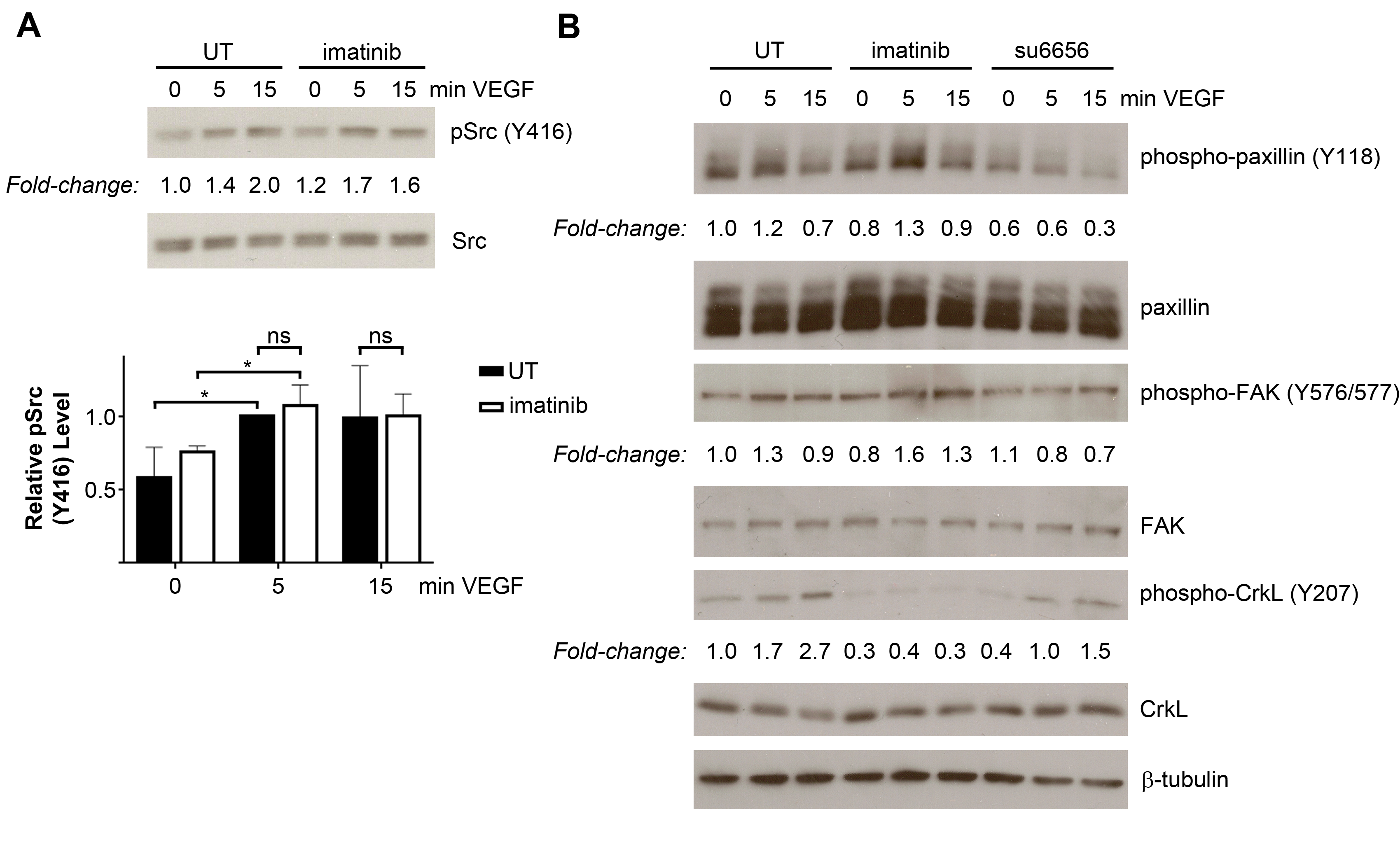

Supplement: Figure S1 — Abl kinase inhibition did not alter VEGF-induced Src activation. (A) Assessment of phospho-Src (Y416) levels in HMVECs treated for the indicated times with 100ng/mL VEGF +/- 10μM imatinib. Phospho-Src (Y416) levels, normalized to total Src levels, are quantified in the bottom panel. Values are presented as means +/- SD (n=3), relative to levels in VEGF-treated cells (5 min). *P<0.05; ns = not significant. (B) Evaluation of VEGF-induced phosphorylation of paxillin (Y118) and FAK (Y576/577) in HMVECs pre-treated with 10μM imatinib, 1μM su6656, or vehicle control (UT). (TIF) [file pone.0085231.s001.tif]

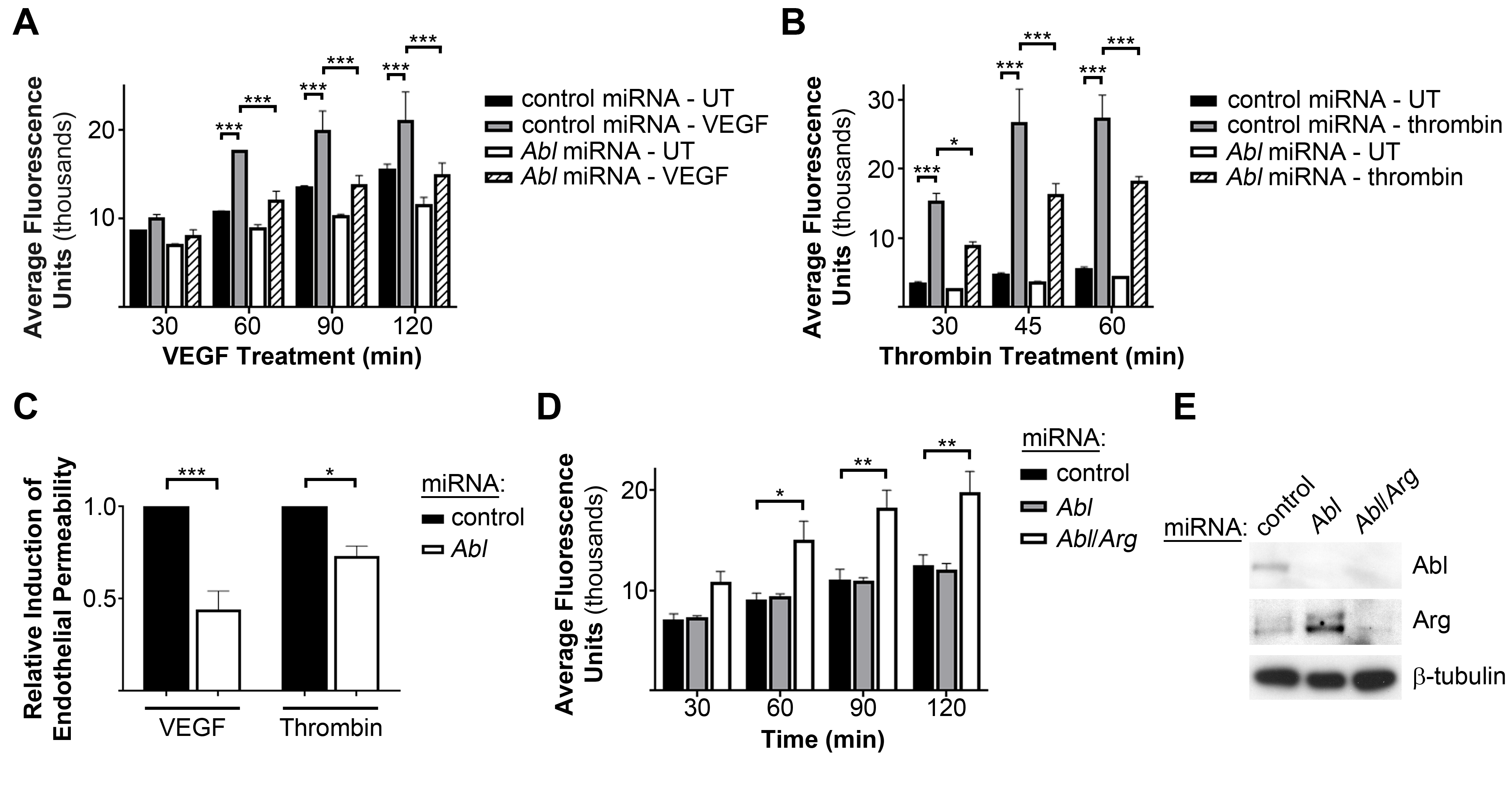

Supplement: Figure S2 — Impaired endothelial permeability following Abl knockdown. (A and B) Assessment of permeability of HMVEC monolayers expressing either control or Abl miRNAs to fluorescein-labeled dextran (molecular weight 40kDa), following (A) VEGF (100ng/mL) or (B) thrombin (1U/mL) treatment. Data shown are mean fluorescence of samples collected from bottom Transwell chambers at the indicated times following VEGF or thrombin treatment, +/- SD of three replicates per treatment. Data are representative of 4-5 independent experiments. (C) Quantification of inhibition of VEGF- and thrombin-induced endothelial permeability following Abl knockdown. Values are expressed relative to permeability of HMVECs expressing control miRNA. Data are presented as means +/- SEM (VEGF, n=5; thrombin, n=4). (D) Evaluation of baseline permeability to fluorescein-labeled dextran of unstimulated HMVEC monolayers expressing control, Abl, or Abl/Arg miRNAs. Data are presented as means +/- SEM (n=4). (E) Assessment of Abl and Arg protein levels in HMVECs following Abl or Abl/Arg knockdown. *P<0.05; **P<0.01; ***P<0.001. (TIF) [file pone.0085231.s002.tif]

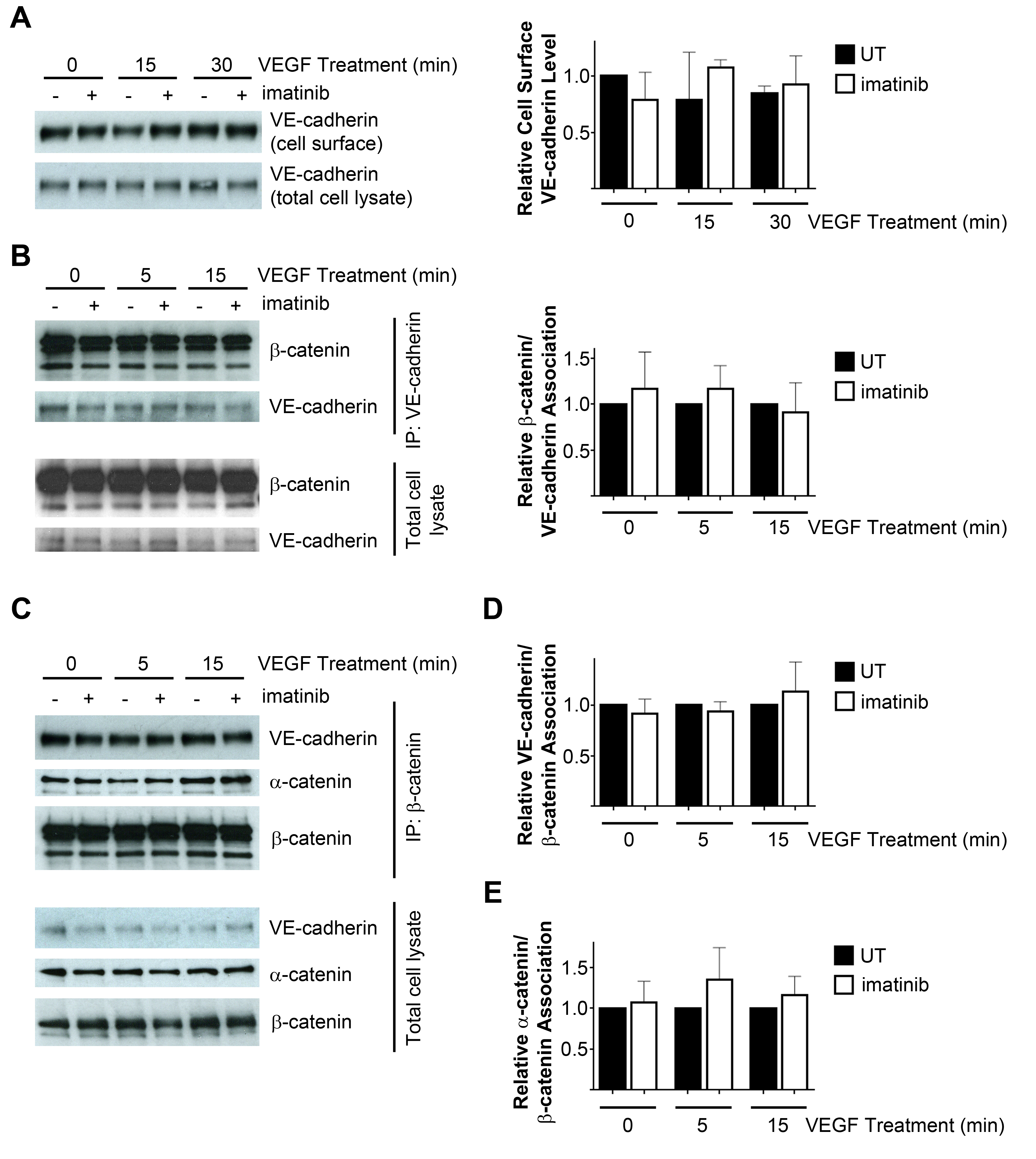

Supplement: Figure S3 — Abl kinase inhibition did not alter VE-cadherin cell surface levels or adherens junction complex association. (A) Evaluation of total and cell surface VE-cadherin protein levels in HMVECs treated with VEGF (100ng/mL) with or without imatinib pre-treatment (10μM), as assessed by biotinylation of cell surface proteins. Cell surface VE-cadherin levels are quantified in the right panel, relative to levels in untreated cells (UT). Data are presented as means +/- SD (n=3). (B) Assessment of VE-cadherin association with β-catenin in HMVECs treated with VEGF +/- imatinib, following VE-cadherin immunoprecipitation. Data are quantified in the right panel as means +/- SD (n=5), relative to co-immunoprecipitated β-catenin levels in vehicle-treated cells (UT) at each time point. (C-E) Assessment of β-catenin association with VE-cadherin and α-catenin in HMVECs treated with VEGF +/- imatinib, following β-catenin immunoprecipitation. (D-E) Quantification of levels of co-immunoprecipitated (D) VE-cadherin and (E) α-catenin, relative to levels in vehicle-treated cells (UT) at each time point. Data are presented as means +/- SD (VE-cadherin, n=5; α-catenin, n=2). (TIF) [file pone.0085231.s003.tif]

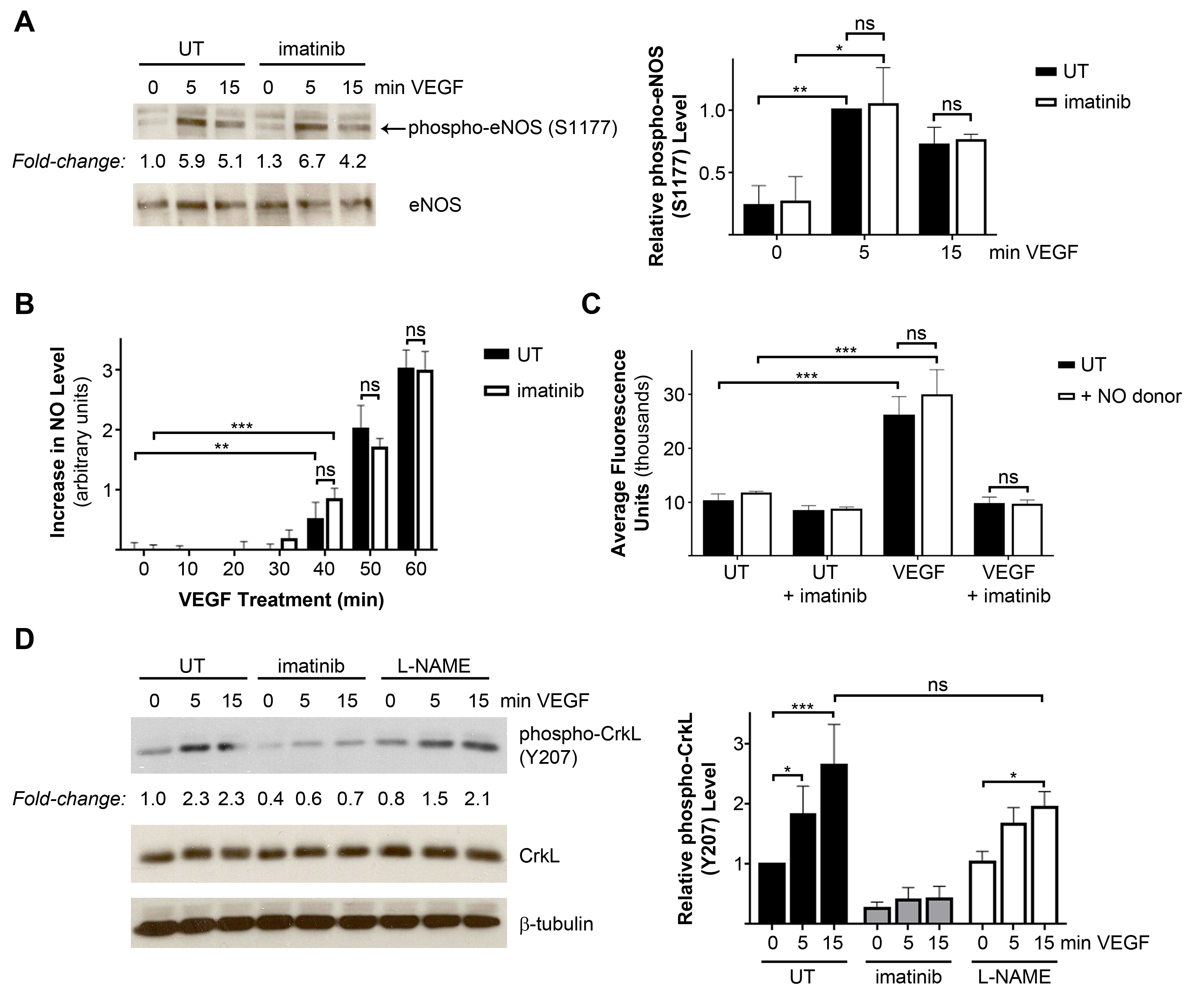

Supplement: Figure S4 — No effect of Abl kinase inhibition on VEGF-induced nitric oxide production. (A) Assessment of eNOS (S1177) phosphorylation in HMVECs following 5 or 15 minutes treatment with 100ng/mL VEGF, in the absence (UT) or presence of 10μM imatinib. Phospho-eNOS (S1177) levels, normalized to total levels, are quantified in the right panel. Values are expressed as means +/- SD (n=3), relative to levels in VEGF-treated cells (5 min). (B) Evaluation of VEGF-induced nitric oxide (NO) production in HMVECs, +/- imatinib, relative to levels in unstimulated cells. Values are expressed as means +/- SD of 4 fields per treatment and are representative of 3 independent experiments. (C) Evaluation of endothelial monolayer permeability, as assessed by passage of fluorescein-labeled dextran (molecular weight 40kDa) through HMVEC monolayers grown on Transwells, following treatment with VEGF (100ng/mL, 60 minutes) with or without imatinib pre-treatment, in the absence (UT) or presence of the NO donor SNAP (100μM). Data shown are mean fluorescence of samples collected from bottom Transwell chambers, +/- SD of three replicates per treatment. Data are representative of three independent experiments. (D) Assessment of Abl kinase activation, as determined by phospho-CrkL tyrosine (Y) 207 levels, following stimulation of serum-starved HMVECs with 100ng/mL VEGF for 5 or 15 minutes, with or without pre-treatment with 10μM imatinib or 200μM L-NAME. pCrkL (Y207) levels (normalized to total CrkL) are quantified in the right panel, relative to levels in untreated (UT) cells. Data are presented as means +/- SD (n=3). *P<0.05; **P<0.01; ***P<0.001; ns = not significant. (TIF) [file pone.0085231.s004.tif]

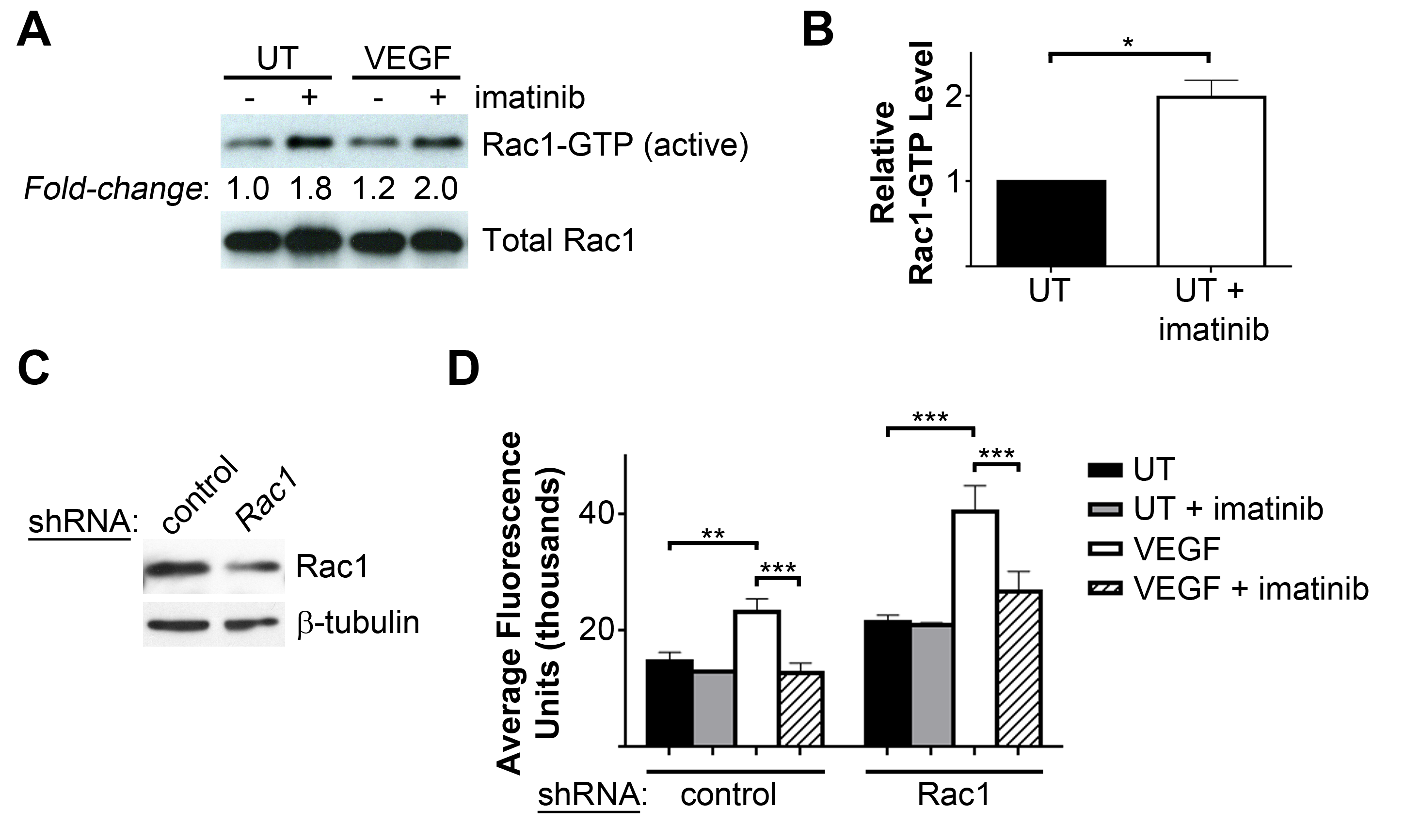

Supplement: Figure S5 — Increased Rac1 GTPase activity following Abl kinase inhibition. (A-B) Assessment of levels of GTP-bound (active) Rac1 GTPase in HMVECs treated with imatinib (10μM), then treated with VEGF (100ng/mL, 2 minutes) or left unstimulated (UT). Rac1-GTP levels, normalized to total Rac1, are quantified in (B), relative to levels in vehicle-treated cells (UT). Data are presented as means +/- SD (n=2). (C) Assessment of Rac1 protein levels following Rac1 shRNA expression. (D) Evaluation of permeability of HMVECs expressing either control or Rac1 shRNAs to fluorescein-labeled dextran, following 60 minutes VEGF stimulation with or without imatinib pre-treatment. Data shown are mean fluorescence of samples collected from bottom Transwell chambers, +/- SD of three replicates per treatment. Data are representative of three independent experiments. *P<0.05; **P<0.01; ***P<0.001. (TIF) [file pone.0085231.s005.tif]

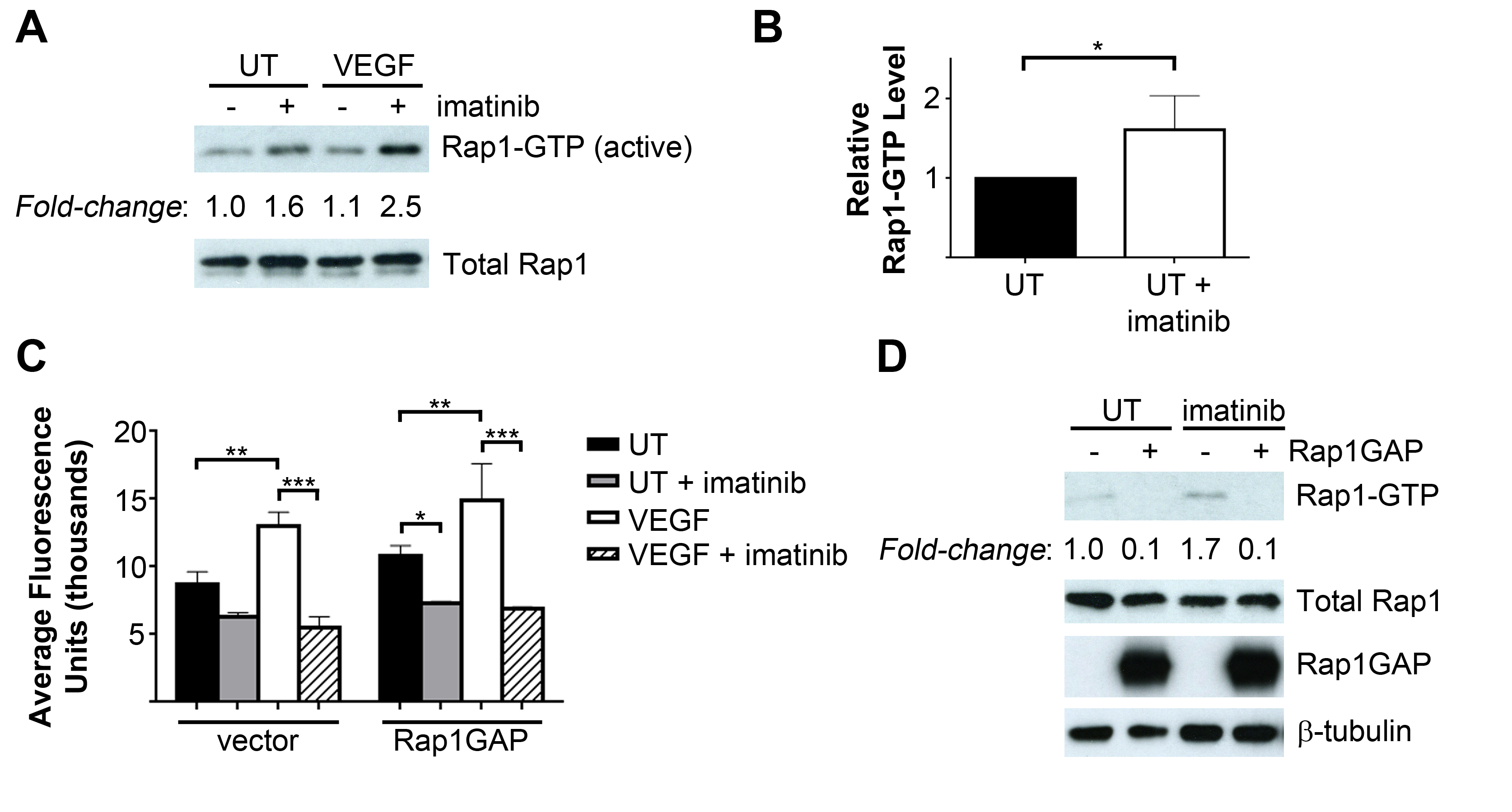

Supplement: Figure S6 — Increased Rap1 GTPase activity following Abl kinase inhibition. (A-B) Assessment of levels of GTP-bound (active) Rap1 GTPase in HMVECs treated with imatinib (10μM), either treated with VEGF (100ng/mL, 2 minutes) or left unstimulated (UT). Rap1-GTP levels, normalized to total Rap1, are quantified in (B), relative to levels in vehicle-treated cells (UT). Data are presented as means +/- SD (n=5). (C) Evaluation of permeability of HMVECs expressing either Rap1GAP or vector control to fluorescein-labeled dextran, following 60 minutes VEGF treatment with or without imatinib pre-treatment. Data shown are mean fluorescence of samples collected from bottom Transwell chambers, +/- SD of three replicates per treatment. Data are representative of two independent experiments. (D) Assessment of levels of active, GTP-bound Rap1 in vehicle (UT)- or imatinib-treated cells expressing either Rap1GAP or vector control. *P<0.05; **P<0.01; ***P<0.001. (TIF) [file pone.0085231.s006.tif]

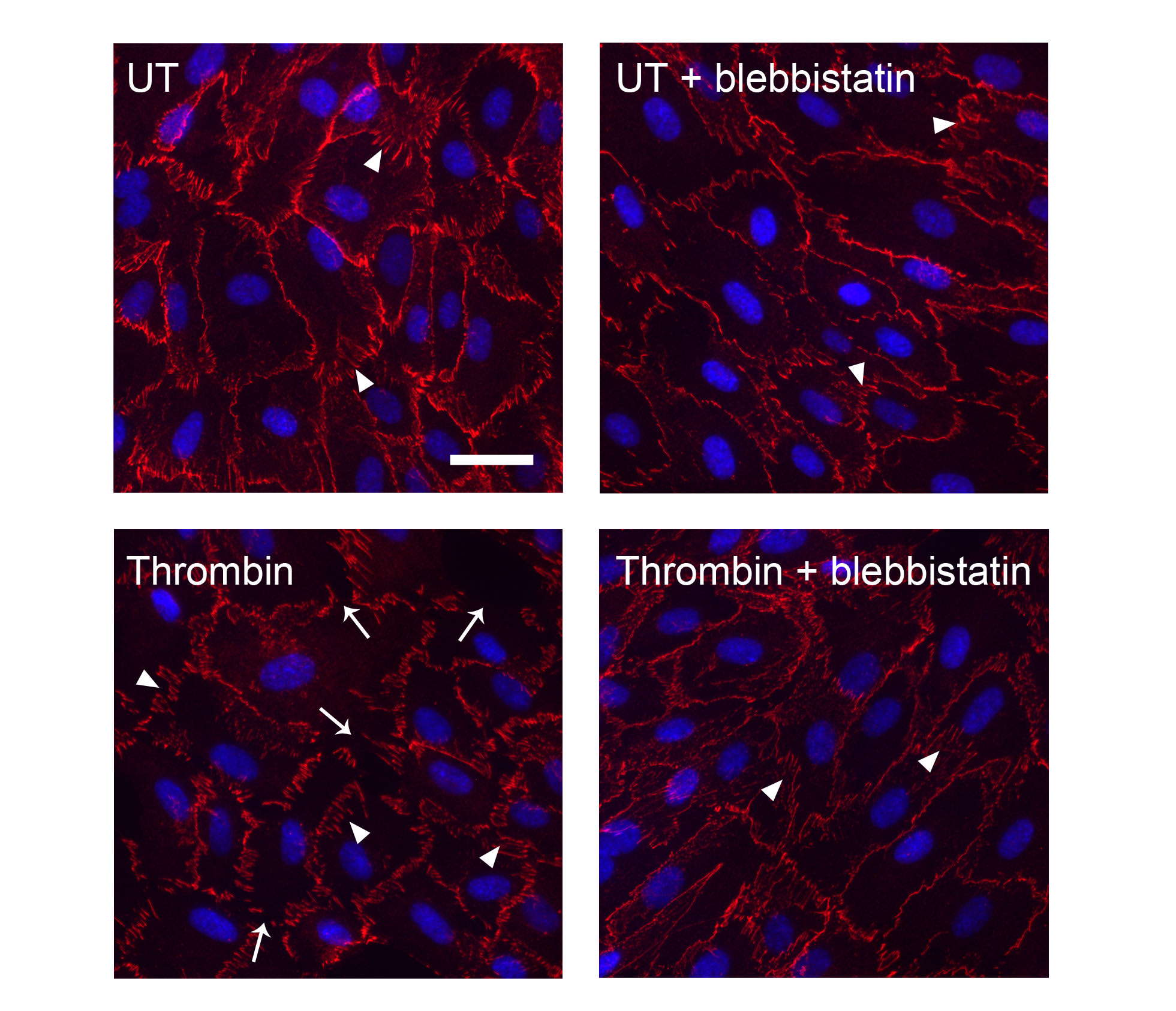

Supplement: Figure S7 — Inhibition of acto-myosin contractility impaired thrombin-induced disruption of endothelial adherens junctions. Staining of HMVEC monolayers for the adherens junction marker VE-cadherin (red) following treatment with thrombin (1U/mL, 5 minutes), with or without pre-treatment with the non-muscle myosin II ATPase inhibitor blebbistatin (5μM). Thrombin treatment induced formation of inter-endothelial cell gaps (arrows) and destabilization of endothelial cell-cell junctions (“zig-zag” VE-cadherin staining pattern, arrowheads), which were reduced by blebbistatin pre-treatment. (TIF) [file pone.0085231.s007.tif]

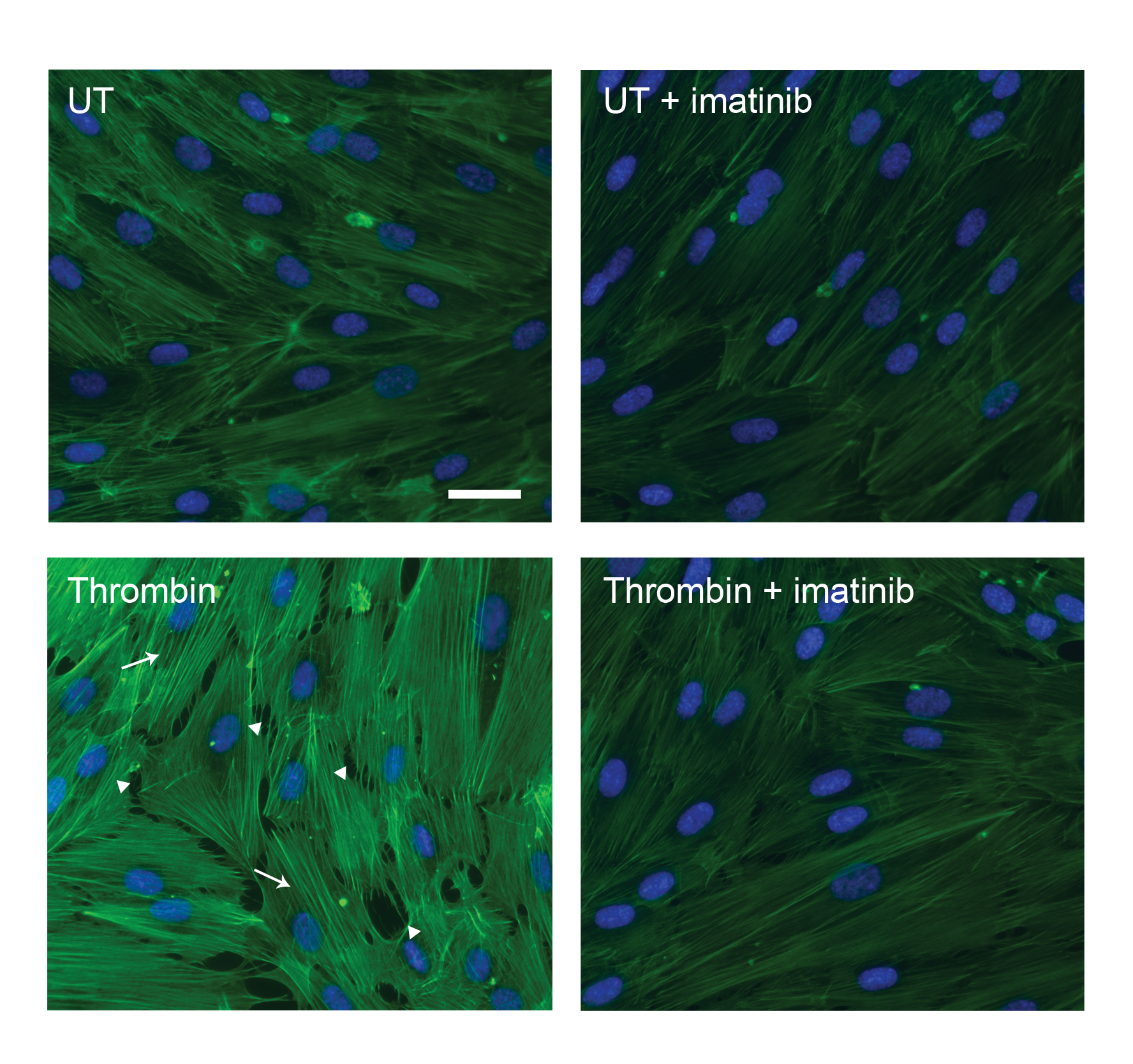

Supplement: Figure S8 — Abl kinase inhibition impaired thrombin-induced formation of actin stress fibers. Evaluation of actin cytoskeletal structure, as assessed by phalloidin staining, in HMVECs treated with thrombin (1U/mL, 5 minutes), either in the absence (UT) or presence of 10μM imatinib. Thrombin treatment resulted in formation of actin stress fibers (arrows) and intercellular gaps (arrowheads), which were inhibited by imatinib pre-treatment. (TIF) [file pone.0085231.s008.tif]

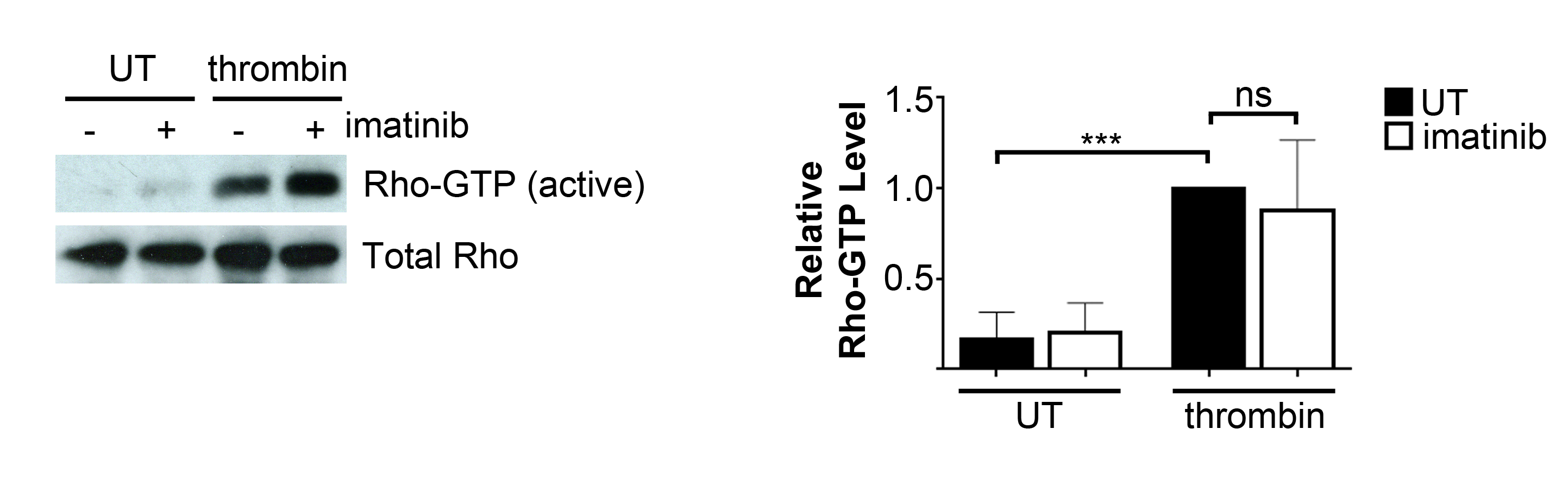

Supplement: Figure S9 — Abl kinase inhibition did not affect thrombin-induced activation of Rho GTPase. Assessment of levels of GTP-bound (active) Rho GTPase in HMVECs either left unstimulated (UT) or treated with thrombin (1U/mL, 2 minutes), +/- imatinib (10μM). Rho-GTP levels, normalized to total Rho, are quantified in the right panel, relative to levels in thrombin-stimulated cells. Data are presented as means +/- SD (n=5). ***P<0.001. (TIF) [file pone.0085231.s009.tif]
